# Vesicular and uncoated Rab1-dependent cargo carriers facilitate ER to Golgi transport

**DOI:** 10.1242/jcs.239814

**Published:** 2020-07-24

**Authors:** Laura M. Westrate, Melissa J. Hoyer, Michael J. Nash, Gia K. Voeltz

**Affiliations:** 1 Department of Chemistry and Biochemistry, Calvin University, Grand Rapids, MI 49546, USA; 2 Howard Hughes Medical Institute, Department of Molecular, Cellular, and Developmental Biology, University of Colorado-Boulder, Boulder, CO 80309, USA; 3 Department of Molecular, Cellular, and Developmental Biology, University of Colorado-Boulder, Boulder, CO 80309, USA

**Keywords:** COPII, Rab1, ERES, TNF-α, MANII, RUSH

## Abstract

Secretory cargo is recognized, concentrated and trafficked from endoplasmic reticulum (ER) exit sites (ERES) to the Golgi. Cargo export from the ER begins when a series of highly conserved COPII coat proteins accumulate at the ER and regulate the formation of cargo-loaded COPII vesicles. In animal cells, capturing live *de novo* cargo trafficking past this point is challenging; it has been difficult to discriminate whether cargo is trafficked to the Golgi in a COPII-coated vesicle. Here, we describe a recently developed live-cell cargo export system that can be synchronously released from ERES to illustrate *de novo* trafficking in animal cells. We found that components of the COPII coat remain associated with the ERES while cargo is extruded into COPII-uncoated, non-ER associated, Rab1 (herein referring to Rab1a or Rab1b)-dependent carriers. Our data suggest that, in animal cells, COPII coat components remain stably associated with the ER at exit sites to generate a specialized compartment, but once cargo is sorted and organized, Rab1 labels these export carriers and facilitates efficient forward trafficking.

This article has an associated First Person interview with the first author of the paper.

## Abstract (precis)

**Highlighted Article:** Live-cell *de novo* cargo trafficking has revealed that secretory cargo is capable of leaving the endoplasmic reticulum in COPII-uncoated Rab1-dependent vesicle carriers.

## INTRODUCTION

The endoplasmic reticulum (ER) serves as the entry point for the secretory pathway. Following successful translation and translocation, secretory proteins synthesized in the ER are concentrated by COPII proteins at ER exit sites (ERES) and trafficked to the Golgi within vesicular carriers (D'Arcangelo et al., 2013). The order of assembly of the COPII coat and its role in generating a vesicle that contains secretory cargo has been dissected in molecular detail by elegant *in vivo* and *in vitro* studies. The major components include a guanine exchange factor, Sec12, that recruits and activates the small GTPase Sar1 to the ER (Nakano and Muramatsu, 1989; Nakano et al., 1988). Upon GTP activation, Sar1 inserts an amphipathic helix into the ER membrane to initiate membrane deformation (Lee et al., 2005). Sar1 next recruits the heterodimer ‘inner coat’ complex Sec23/Sec24, which stabilizes membrane curvature and recruits cargo through the cargo binding sites on Sec24 (Bi et al., 2002; Kuehn et al., 1998; Miller et al., 2002, 2003). The inner coat recruits the heterotetramer ‘outer coat’ complex composed of Sec13/Sec31, resulting in the formation of a fully formed COPII-coated transport vesicle that delivers incorporated cargo to the Golgi complex via anterograde trafficking from the ER (Kirk and Ward, 2007; Lederkremer et al., 2001; Lee et al., 2004; Rossanese et al., 1999; Stagg et al., 2006). Many of these studies have been performed in yeast cells because of the advantages of yeast genetics in identifying COPII components, combined with temperature-sensitive mutants that can stall cargo as it is trafficked.

Animal cells may have adapted alternative methods to ensure rapid cytoplasmic trafficking of cargo from ERES to the Golgi in a vast cytoplasm. Early reports on mammalian cells suggested that COPII vesicles rapidly lose their coat after scission from the ER (Antonny et al., 2001; Aridor et al., 1995; Scales et al., 1997; Presley et al., 1997; Stephens et al., 2000) and immuno-electron microscopy evidence identified the existence of free COPII-coated vesicles at the ER–Golgi interface (Zeuschner et al., 2006). *De novo* ER to Golgi cargo trafficking is difficult to resolve in live animal cells because secretory proteins are continuously being synthesized and move rapidly through the secretory pathway. Additionally, temperature-sensitive mutants of the COPII coat are not available in animal cells. Instead, although various techniques have been used to study secretory cargo trafficking in animal cells, the majority of studies have utilized a single temperature-sensitive fluorescently tagged cargo, ts-VSVG (Bonfanti et al., 1998; Gallione and Rose, 1983; Kreitzer et al., 2000; Shomron et al., 2019 preprint). At non-permissive temperature, GFP-ts-VSVG is stuck in the ER as an unfolded protein. A shift to permissive temperature allows VSVG to fold and be exported. This cargo can be visualized live as it leaves the ER in animal cells, and previous experiments with this cargo have suggested that although the cargo initially co-localizes with a component of the COPII coat at ERES, it does not exit the ERES with Sec24 (Presley et al., 1997; Stephens et al., 2000). These data first suggested that some aspects of the transition from COPII-coated vesicle formation to a vesicular structure trafficking towards the Golgi might not require COPII.

The current model for mammalian ER to Golgi trafficking remains somewhat controversial. Many have cited that ER to Golgi trafficking relies on both vesicular and tubular intermediate compartments (Saraste and Svensson, 1991; Stinchcombe et al., 1995; Watson and Stephens, 2005; Xu and Hay, 2004). The proposed function of these structures is to sort the ER exit site cargo to the Golgi (Bannykh et al., 1996). Many of these structures are not static and can make long-range movements (Presley et al., 1997; Scales et al., 1997). Studies monitoring the VSVG cargo system have shown that when the temperature is reduced, intermediate structures become enlarged, accumulate secretory cargo and form in the vicinity of COPII-labeled ERES (Mironov et al., 2003; Presley et al., 1997; Stephens, 2003). These structures acquire the vesicular coat complex COPI (Aridor et al., 1995; Lee et al., 2004; Shomron et al., 2019 preprint; Stephens et al., 2000) and, upon temperature release, the structures move towards the Golgi complex in a microtubule-dependent manner (Presley et al., 1997; Shima et al., 1999; Stephens et al., 2000). More recent studies have keyed in on how these intermediate compartment structures rearrange to facilitate trafficking of large cargoes such as collagen (McCaughey et al., 2019; Saito et al., 2009). With the help of Tango1, intermediate structures could potentially form tunnels from the ER to Golgi (Raote and Malhotra, 2019). It is currently unclear whether the creation of larger tubular clusters is due to large cargoes or, potentially, an abundance of cargo in cells. However, with the advent of faster and more sensitive imaging techniques and the development of newly trackable trafficking cargoes, we can now combine these advanced methods to visualize this vital cargo trafficking step in live cells.

Here, we follow trafficking of COPII ERES markers and ER export cargoes, including the classically used ts-VSVG and two cargoes with varying size that release from the ER upon addition of biotin using the ‘retention using selective hooks’ (RUSH) system (Boncompain et al., 2012; Presley et al., 1997). We have optimized the RUSH system to measure the rapid release of *de novo* secretory cargo trafficking from ERES relative to the COPII coat components and the Rab1 GTPase (herein referring to Rab1a or Rab1b). We have used three different mammalian cell lines, four fluorescently tagged COPII coat proteins, two different cargoes and a Rab membrane marker to demonstrate that COPII coat components predominately remain at stable ERES as cargo leaves in a Rab1-dependent carrier.

## RESULTS

### COPII components localize to stable domains on peripheral ER tubules

**Fig. 1.**
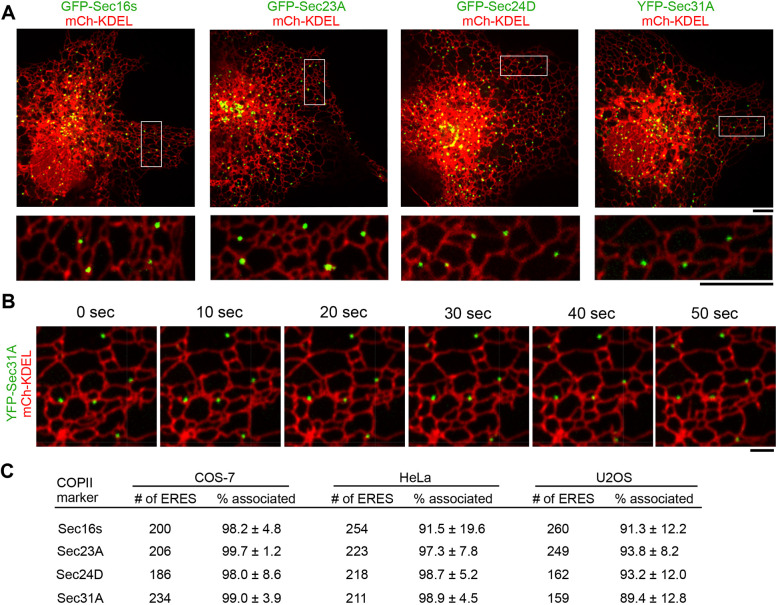
**COPII components localize to stable domains on peripheral ER tubules.** (A) Representative merged images of COS-7 cells reveal the distribution of the ER membrane network relative to COPII components by live cell confocal fluorescence microscopy (ER labeled with mCh-KDEL in red; GFP-Sec16s, GFP-Sec23A, GFP-Sec24D, or YFP-Sec31A in green). Magnification of boxed areas shown below. (B) COPII outer coat component YFP-Sec31A (green) is tracked over time for 2 min to quantify YFP-Sec31A puncta association with the ER (mCh-KDEL, red) over time. (C) COPII-labeled puncta (as in B) were tracked live over time to determine whether they remain tightly associated with the ER network for the duration of the 2 min movie in COS-7, HeLa, or U2OS cells. 3 replicates; 15 cells for Sec16s, 15 cells for Sec23A, 19 cells for Sec24D and 18 cells for Sec31A. Scale bars: 5 µm (A), 2 µm (B).

We used live confocal fluorescence microscopy to visualize the distribution and dynamics of four major COPII components at ERES in three different animal cell lines: COS-7, HeLa and U2OS. First, we assessed the location and dynamics of fluorescently tagged and exogenously expressed GFP-Sec16s, a resident ER protein that serves as a scaffold for COPII assembly at ERES (Watson et al., 2006). GFP-Sec16s localized to punctate structures along ER tubules in all three cell types (Fig. 1A; Fig. S1, Movie 1). We collected 2 min movies (with 5 s intervals) of GFP-Sec16s puncta to track their location relative to the tubular ER network [labeled with mCherry (mCh)-KDEL] over time. As expected for an ERES scaffold, the vast majority of Sec16s puncta (98.2%) remained associated with the ER network throughout the length of the movie (puncta were scored as ‘associated’ if they were visible and tracked with an ER tubule for the entire length of the movie).

We performed a similar analysis on fluorescently tagged COPII inner and outer coat components Sec23A, Sec24D and Sec31A (Fig. 1A). Although most of the COPII mammalian isoforms are functionally redundant, the four mammalian Sec24 isoforms have distinct and overlapping specificity in the cargo export signals recognized (Khoriaty et al., 2018; Stankewich, 2006; Wendeler et al., 2007). Sec24D was chosen because it binds strongly to four out of six common ER export signals, whereas the other three Sec24 isoforms strongly bind only two out of six (Wendeler et al., 2007). Similar to GFP-Sec16s, puncta of GFP-Sec23A, GFP-Sec24D and YFP-Sec31A were all distributed throughout the cytoplasm and were all tightly associated with the tubular ER network during 2 min movies and in multiple cell types (Fig. 1B,C; Fig. S1, Movies 2-4). Nearly 100% of all COPII components remained associated with the ER throughout the length of the movie (98.2±4.8% for Sec16s, 99.7±1.2% for Sec23A, 98.0±8.6% for Sec24D and 99.0±3.9% for Sec31A in COS-7 cells; Fig. 1C). COPII components were scored as remaining associated with the ER if they remained tethered to the ER throughout the 2 min movie. The small percentage of COPII components that were marked as not being associated with the ER often represented puncta that appeared to become dissociated from the ER as a result of the ER network or a COPII punctum moving out of the focal plane. Thus, despite being associated with the ER for the majority of the movie, if there were at least two sequential frames (10 s) where the ER and COPII punctum could not be tracked together, the COPII component was marked as ‘not associated.’ HeLa and U2OS cells, which are thicker than COS7 cells, showed a higher propensity for non-association and therefore demonstrated slightly reduced percentages of COPII components remaining associated with the ER through the 2 min movie (Fig. 1C). Notably, we never captured a COPII-coated vesicle budding off or trafficking away from the ER in live cell images. These data suggest that COPII coat components form stable structures at ERES in animal cells.

To challenge the strength of the association between the ER and COPII-coated domains, we treated cells with ionomycin, an ionophore that triggers increased intracellular calcium and subsequent fragmentation of the ER (Fig. S2A) (Koch et al., 1988). COS-7 cells were treated with 2 µM ionomycin (5 min) and fixed before acquiring confocal z-slices through the entire cell. Even upon fragmentation of the ER, we still observed that COPII-labeled domains remained co-localized with ER vesicles: 86.7±13.6% for Sec16s, 91.3±10.2% for Sec23A, 94.1±6.8% for Sec24D and 89.3±15.3% for Sec31A (Fig. S2B-D). These data further support the notion that COPII domains are integral components of ERES in animal cells.

Further evidence that COPII components form stable structures at ERES in animal cells came from fluorescent recovery assays used to measure the dynamics of the COPII outer coat component Sec31A in COS7 and HeLa cells (Fig. S3A,B, respectively). A 10×10 µm region of interest (ROI) was photobleached in the peripheral ER of cells expressing outer coat component YFP-Sec31A and a general ER marker (mCh-KDEL). The YFP signal within the ROI was hit with a high-power 488 nm laser to diminish YFP signal selectively. We intentionally picked a laser exposure that did not result in 100% loss of signal so that we could confidently track whether fluorescence recovered to the ROI that marked the original COPII site on the ER (Fig. S3C). Fluorescence intensity was calculated at the frame immediately before photobleaching (pre), the frame immediately after bleaching (post) and 30 s after bleaching. In both COS-7 and HeLa cells, fluorescence recovery occurred at the ROI marking the original COPII puncta nearly 90% of the time (87.9±6.53% in COS7, 88.8±4.25% in HeLa cells; Fig. S3D) suggesting that COPII components are recruited to stable ERES domains.

### Dynamic COPII domains are tethered to sliding ER tubules

**Fig. 2.**
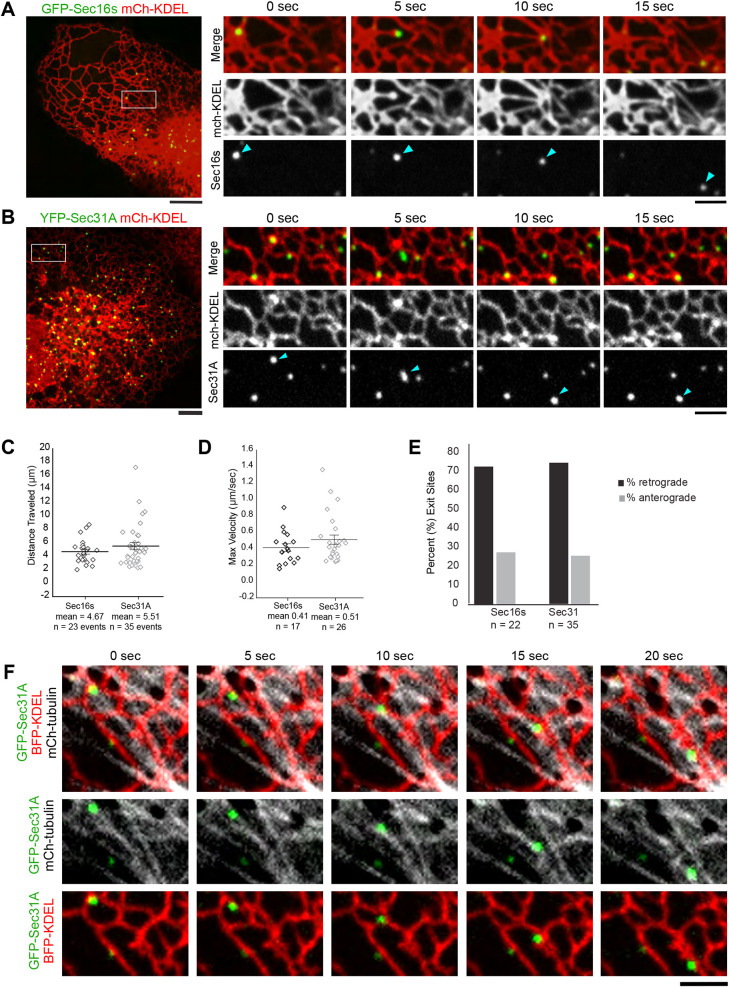
**Dynamic COPII domains are tethered to sliding ER tubules.** (A) Representative image of a COS-7 cell expressing an ER marker (mCh-KDEL in red) and GFP-Sec16s puncta (in green). In the zoomed insets, the Sec16s puncta (arrowheads) tracks with an attached ER tubule over time. (B) As in A for YFP-Sec31A puncta dynamics. (C) Total distance traveled by individual COPII-labeled domains displays long range movement within the cell (9 cells and 23 exit sites for Sec16s; 11 cells and 35 events for Sec31A). (D) Maximum velocity of indicated COPII puncta dynamics during long range movements. Maximum velocity was calculated by determining the maximum distance traveled between sequential frames acquired 5 s apart (9 cells and 17 events for Sec16s; 11 cells and 26 events for Sec31A). Error bars represent s.e.m. (E) Percentage of dynamic Sec16s or Sec31 puncta from C that moved in the retrograde versus anterograde direction. (F) Representative example of Sec31A punctum moving along an established microtubule while still associated with the ER. COS-7 cells expressed mCh-tubulin (gray), YFP-Sec31A (green) and BFP-KDEL (red). Scale bars: 5 µm (A,B), 2 µm (insets in A,B; F).

COPII- and ERES-labeled structures have been shown to be relatively stable in yeast and plants (DaSilva et al., 2004; Hanton et al., 2009; Kirk and Ward, 2007; Shindiapina and Barlowe, 2010). To characterize the dynamics of COPII coat components in mammalian cells, we tracked COPII-labeled domains over time. We found that COPII coat components remain tightly associated with the ER. Consistent with previous experiments performed on Sec24D, COPII sites were relatively stable in the peripheral ER (Gupta et al., 2008; Stephens et al., 2000). However, we occasionally observed a COPII-labeled domain traveling a long distance (over 2 µm; Fig. 2A,B) while maintaining contact with ER tubules. The total length of movement was highly variable, with the average distance traveled being about 5 µm (4.67±0.4 µm for Sec16s and 5.51±0.5 µm for Sec31A; Fig. 2C). This type of coupled movement was reminiscent of ER sliding, whereby ER tubules extend along the side of an existing microtubule at a velocity of ∼0.5 µm/s (Friedman et al., 2010). To confirm this mechanism of movement, we measured the maximum velocity of COPII puncta during long-range trajectories. Consistent with known velocities for ER sliding (Friedman et al., 2010), dynamic ER-associated COPII domains traveling more than 2 µm had a maximum velocity of ∼0.5 µm/s along microtubules (0.41±0.03 µm/s for Sec16s and 0.51±0.04 µm/s for Sec31A; Fig. 2D). Additionally, when we observed these dynamic events in conjunction with fluorescently labeled microtubules, we could track COPII puncta traveling along existing microtubules (Fig. 2F), thus providing additional evidence that these long-range movements are trafficking via ER sliding. By tracking the direction of these long-range COPII movements, we found that the majority of events (73% for Sec16s and 74% for Sec31A) move in the retrograde direction towards the Golgi (Fig. 2E). Despite the distance traveled, these dynamic COPII-labeled domains remain tethered to the ER during long-range movements, suggesting that they are not released from the ER membrane.

### Using the RUSH system to monitor cargo trafficking from ER to Golgi

**Fig. 3.**
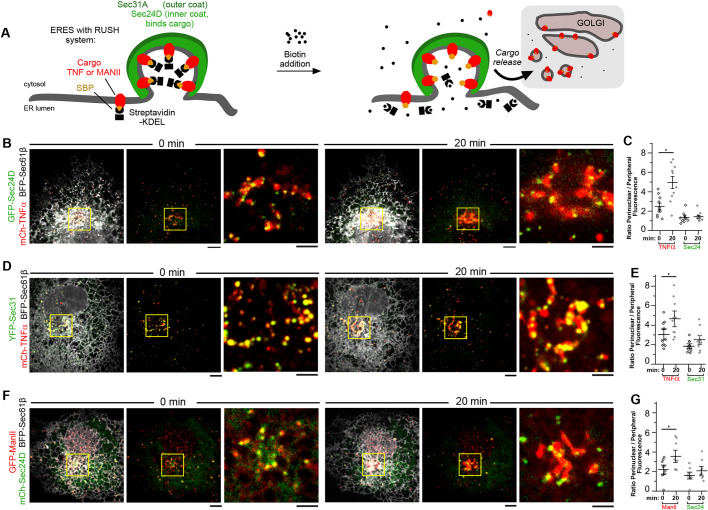
**Using the RUSH system to monitor cargo trafficking from ER to Golgi.** (A) Cartoon of the RUSH system for cargo release from ERES upon biotin addition. (B) Representative image of cargo distribution in a COS-7 cell expressing mCh-TNF RUSH (cargo, red), GFP-Sec24D (COPII, green) and BFP-Sec61β (ER, gray) prior to biotin addition (0 min) and after biotin addition (20 min). Note that at 0 min, the ER network and ERES and TNF cargo are spread throughout the cytoplasm, with ERES and TNF cargo localized to the ER network. By comparison, 20 min after biotin addition, a dramatic increase in TNF cargo accumulation was observed in the perinuclear box (yellow). (C) Quantification of TNF cargo and Sec24D fluorescence in perinuclear box (representative of Golgi region) compared with peripheral signal before (0 min) or after biotin addition (20 min). The 5×5 μm^2^ regions selected in the periphery and perinuclear region were used to calculate fluorescence intensity in those cellular regions. The ratio of perinuclear to peripheral fluorescence signal was used to track any re-distribution of cargo or Sec24D-marked COPII exit sites following biotin addition (*P=0.003). (D,E) As in B and C for ER (BFP-Sec61B), mCh-TNF cargo and the outer coat component YFP-Sec31 (*P=0.024). (F,G) As in B and C for ER (BFP-Sec61B), mCh-Sec24D and GFP-ManII cargo (*P=0.019). Error bars represent s.e.m. Scale bars: 5 µm (B,D,F); 2 µm (insets in B,D,F).

**Fig. 4.**
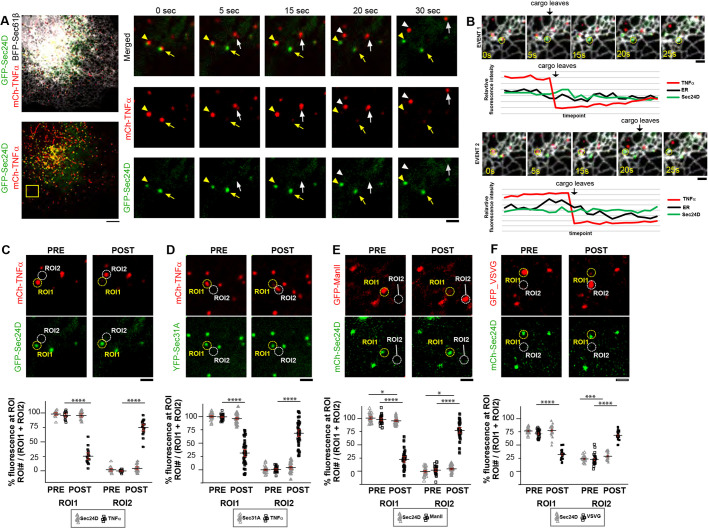
**COPII components remain associated with the ER as cargo is exported.** (A) COS-7 cell expressing mCh-TNF RUSH (red), GFP-Sec24D (green) and BFP-Sec61β (gray). Right: Time-lapse image of two events (from within yellow box) where fluorescently marked cargo (red) is observed leaving COPII fluorescent puncta (green) on the ER (gray) at the indicated time points following biotin addition. Arrows mark the first trafficking event and the arrowheads mark a second event. COPII is marked with a yellow arrow/head in each frame and the location of cargo as it traffics is marked with white. (B) Sec24D, TNF cargo and ER fluorescence was monitored over 2 min within an ROI (yellow circle) that marks the original site of cargo release. Graphs plot the relative fluorescence intensity and reveal that the ER and Sec24D fluorescence levels remain in the circle even after the cargo leaves. (C) As in A, for several events, the coat and cargo fluorescence was measured in pre and and post cargo leaving frames. Circles mark where fluorescence measurements were made. ROI1 is the site from which cargo is released and ROI2 is the site where cargo traffics to. Fluorescence at each region for each marker was background subtracted and the percentage fluorescence at each ROI calculated pre and post cargo leaving (****P<0.0001). (D) As in C for TNF cargo and YFP-Sec31A (****P<0.0001). (E) As in C for GFP-ManII cargo (****P<0.0001) and mCh-Sec24D (*P=0.03). (F) As in C for GFP-VSVG (****P<0.0001) and mCh-Sec24D (***P=0.0004 in ROI2). Error bars represent s.e.m. Scale bars: 5 µm (A); 1 µm (insets in A; B-F).

Next, we analyzed whether COPII-labeled puncta bud off from the ER only once they are loaded with cargo to be trafficked. Conflicting results from the mammalian field have questioned the fate of COPII components following cargo packaging at the ER. Specifically, work by Stephens et al. (2000), Scales et al. (1997) and Presley et al. (1997) using a temperature block cargo release system (VSVG) showed that cargo can be transported to the Golgi in the absence of COPII components, whereas immuno-electron tomography work by Zeuschner et al. (2006) identified the existence of COPII-coated transport vesicles at the ER–Golgi interface. We therefore vetted potential systems for visualizing cargo release from the ERES relative to COPII coat components and ER that would provide a systematic analysis of secretory cargo export dynamics from the ER. We first optimized the RUSH system to synchronize and track the release of cargoes exiting the ER in real time (Fig. 3A) (Boncompain et al., 2012). The RUSH system utilizes an ER ‘hook’ protein fused to streptavidin and a secretory cargo protein fused to streptavidin-binding protein (SBP), which under normal conditions traps the secretory cargo in the ER as a result of the streptavidin–SBP interaction. Biotin addition outcompetes the streptavidin–SBP interaction, resulting in synchronous release of secretory cargo from the ER that can be tracked over time. SBP was linked to two different fluorescently tagged cargoes for comparison: TNFα-SBP-mCh and ManII-SBP-GFP. The expression of TNFα-SBP-mCh or ManlI-SBP-GFP resulted in a striking localization pattern where the cargo, while localized to the ER (0 min in Fig. 3), was observed to be significantly enriched in small puncta reminiscent of what we see when we label for COPII proteins. Cargo concentration at ERES has been demonstrated to be an early step in the secretory pathway, with subsequent quality control checkpoints at the ER to ensure that only properly folded proteins are allowed to traffic to vesicular-tubular clusters and the trans-Golgi network (Dukhovny et al., 2008, 2009; Mezzacasa and Helenius, 2002). We therefore predicted that these small puncta represent concentrated cargo at COPII sites that was unable to undergo forward transport because of its association with the streptavidin ER hook protein. Dual labeling of both fluorescently tagged inner and outer COPII coat proteins (GFP-Sec24D and YFP-Sec31A, respectively) with the cargo revealed that these cargo-enriched puncta are often co-labeled with COPII proteins (Fig. 3B,D,F; Fig. 4). This implies that, in the absence of biotin, the RUSH cargo is still capable of being loaded into COPII-marked exit sites that are stalled for export as a result of the streptavidin–SBP interaction occurring between the cargo and ER hook protein.

Biotin addition released forward trafficking of these SBP-tagged secretory cargoes and TNFα-SBP-mCh became enriched at the Golgi within 20 min of biotin treatment (Fig. 3B). By comparison, GFP-Sec24D (Fig. 3B,C) or YFP-Sec31A (Fig. 3D,E) coat components remained at puncta associated with the ER network and did not accumulate with cargo at the Golgi. Similar results were seen with a different cargo, ManII-SBP-GFP, which also accumulated at the Golgi within 20 min of biotin addition, whereas again the mCh-Sec24D coat component, even though it directly binds cargo during initial cargo sorting, also remained in ER-associated puncta (Fig. 3F,G). Significant redistribution of cargo to the perinuclear region of the cell following biotin addition was noted for mCh-TNFα and GPF-ManII cargo (*P*=0.003, 0.024 and 0.019 in Fig. 3C, E and G, respectively). These data show that it is possible to stall and then release cargo in order to visualize how cargo traffics to the Golgi relative to COPII coat components.

### COPII components remain linked to the ER while cargo is exported away

Next, we used the RUSH system to visualize cargo release from ERES and further investigate the properties of cargoes upon ER release and trafficking to the Golgi. First, we stalled TNFα-SBP-mCh at Sec24D-labeled ERES and labeled the ER with BFP-Sec61β (Fig. 4A,B). Following biotin addition, we visualized TNFα-SBP-mCh cargo as it trafficked away from the ER in punctate carriers. Strikingly, fluorescence associated with the GFP-Sec24D puncta stayed associated with the peripheral ER and did not track with the movement of the cargo (Fig. 4A, compare yellow and white arrows; Movie 5). Cargo carriers dissociated from the tubular ER network as they trafficked (Fig. 4B). To quantify the amount of TNF cargo relative to the amount of COPII coat protein that traffics away from the ER, we measured the fluorescence intensity of the ER, TNFα-SBP-mCh cargo and Sec24D in a region of interest (ROI) (Fig. 4B, yellow circle) surrounding the ERES where cargo is released from the ER. Fluorescence intensity at this site was plotted over a 2 min time course (Fig. 4B). We measured a drop in relative fluorescence for TNFα-SBP-mCh signal in the ERES region when cargo left the site. By comparison, relative fluorescent intensity for the COPII coat marker GFP-Sec24D and the ER signal BFP-Sec61β both remained constant before and after cargo release (Fig. 4B, line scan analysis depicting fluorescence at the site at each time point). These data suggest that cargo separates from COPII coat components as it exits the ER to traffic to the Golgi.

To obtain a more quantitative picture of cargo trafficking from COPII components at ERES, we captured further events where cargo is exported from the ER. For each event, we analyzed the cargo leaving frames just before (pre) and just after (post) the event. In these frames, we defined equal area ROIs encircling where cargo initiated from in the pre frame (ROI1) and encircled where cargo trafficked to in the post frame (ROI2) (Fig. 4C). A dramatic drop in percentage fluorescence was measured from pre to post frames for the ROI1 location for TNFα-SBP-mCh signal but not for the GFP-Sec24D signal in these events (*P*<0.0001; Fig. 4C). To further confirm that we saw cargo leaving from the ER without the COPII coat, we performed the same analyses done for the TNFα-SBP-mCh cargo with the Sec24D COPII coat system, but varied the COPII coat marker (Sec31A) (Fig. 4D) and the type of RUSH cargo (ManII-SBP-GFP) (Fig. 4E). Additionally, we monitored the ts-VSVG system with the Sec24D COPII coat (Fig. 4F). As with the TNFα-SBP-mCh/Sec24D system, the TNFα-SBP-mCh cargo left the Sec31A-marked ERES, ManII-SBP-GFP cargo left the Sec24D-marked ERES and VSVG cargo left the Sec24D-marked ERES (Fig. 4D-F; *P*<0.0001 for each condition except for pre/post analysis of Sec24D in Fig. 4E where *P*=0.03, and the pre/post analysis of Sec24D in Fig. 4F where *P*=0.0004, calculated by comparing cargo fluorescence pre and post event; Fig. S4). In all examples where cargo left ROI1 to traffic to ROI2, the COPII coat components remained at ROI1 and did not travel with cargo to ROI2 (Fig. 4C-F; Fig. S4). Together, these data support a model whereby the COPII coat proteins mark stable sites of protein export where cargo traffics from the ER towards the Golgi in an uncoated carrier. Interestingly, we observed a statistically significant but minimal redistribution of Sec24 into ROI2 following ManII and VSVG cargo release (*P*=0.03; Fig. 4E). This could be the result of combined COPII/cargo movement being captured in ROI2 before complete cargo export had occurred or it could represent dynamic uncoating of the COPII coat being captured following cargo export (Fig. S4).

The cargo that we visualized trafficking from the ER membrane in most cases did not have a tubular structure and usually maintained a circular nature more indicative of a vesicle (see examples in Fig. 4 and Fig. S4). However, due to the resolution of fluorescence microscopy, we could not determine whether these structures were vesicles or a cluster of vesicles in living cells. If these structures were rapidly uncoating, however, one would predict that the fluorescence intensity of COPII components at puncta would be significantly reduced as the cargo was exported from the ER. Instead, we observed negligible loss of COPII fluorescence from the point of cargo export (Fig. 4), suggesting instead that COPII forms stable sites on the ER that do not traffic with cargo as it exports from the ER.

### Rapid time-lapse imaging reveals cargo exports from ER in a carrier uncoated by COPII

**Fig. 5.**
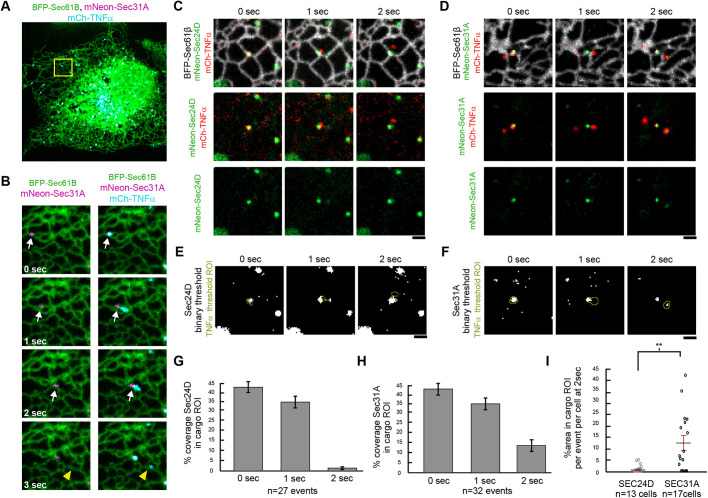
**Rapid time-lapse imaging of release suggests cargo is extruded not uncoated.** (A) Representative example of COS-7 cell expressing BFP-Sec61β, mNeon-31A and mCh-TNFα. (B) Time-lapse images of zoomed inset (yellow box in A) demonstrating COPII and cargo dynamics with 1 s time frames. White arrows track combined movement of ER associated cargo and COPII components. The yellow arrowhead tracks release of cargo from the COPII component. (C,D) Post biotin cargo release event in a COS-7 cell expressing BFP- Sec61β, mCh-TNF RUSH (red) and either mNeon-Sec31A (green; C) or mNeon-Sec24D (green; D). (E,F) Cargo signal was thresholded and converted to binary to create the cargo ROI (yellow outline). To capture any low signal of COPII/Sec protein fluorescence leaving the ER, the Sec31A or Sec24D images were also thresholded and converted to binary. (G) Percentage of Sec24D coverage in the cargo ROI was calculated at 0, 1 and 2 s. (H) As in G but for Sec31A coverage. (I) For the 2 s time point where cargo has completely moved from the ER, the percentage coverage of the cargo ROI per event was measured and this was further averaged per cell to compare Sec24D with Sec31A (1.2%, 13 cells, 27 events versus 12.3%, 17 cells, 32 events), Student's *t*-test (**P=0.003). Error bars represent s.e.m. Scale bars: 1 µm.

To better ascertain the dynamics of COPII components on cargo domains being exported from the ER, we decided to track COPII and cargo export at more rapid time points (Fig. 5; Fig. S5). First, we imaged TNF cargo export relative to Sec24 or Sec31 coat components at a higher frame rate (every 500 ms) to improve visualization of COPII coat and cargo dynamics (Fig. S5A,B). In previous studies, time-lapse images tracking temperature-sensitive cargoes had been performed using 1 s intervals (Stephens and Pepperkok, 2002). As before, the majority of COPII coat fluorescent signal remained behind for both Sec24D (inner coat, cargo binding component) and Sec31A (outer coat) following cargo extrusion (Fig. S5). In all Sec24D events, no coat signal could be captured leaving with the cargo (Fig. S5A). Interestingly, at this high frame rate we could capture a small amount of Sec31A following the cargo signal (Fig. S5B). This fluorescence signal was very dim and asymmetrically distributed, marking only a small percentage of the entire cargo-containing puncta. In addition, it was lost within 5 s of cargo leaving (see images at *t*=8 s in event 1 and *t*=31 s in event 2, Fig. S5B). The rapid loss of the Sec31A fluorescence signal along with the absence of detectable Sec24D signal suggests that cargo is largely being exported from the ER in a carrier domain independent of COPII components.

To confirm that these events leave the ER and were not potentially ER exit sites dividing into two separate sites, we repeated the cargo release experiments, imaging the ER (not previously done by Stephens et al., 2000) in relation to cargo release events (Fig. 5). Even with the addition of the third channel to capture the ER, we were still able to image cargo release events at a high-speed interval (1 s). We sought to dissect further the steps that occurred before and after cargo release in order to measure accurately Sec24D and Sec31A coverage on the cargo puncta that trafficked away from the ER towards the Golgi. For consistency and to directly compare and contrast the behavior of these two COPII components, both Sec24D and Sec31A were tagged with the mNeon fluorophore (Fig. 5).

We first characterized ERES before cargo release. We were able to capture events where the ER, cargo and components moved together as the ER network rearranged pre cargo release (Fig. 5A,B). These events demonstrated that cargo was still linked to the ER just prior to cargo release. All three components (ER, COPII and cargo) first moved together pre cargo release (0-2 s, white arrow, Fig. 5B). Cargo release from ERES was observed at 3 s and was not affiliated with COPII coat components (yellow arrowhead, Fig. 5B). The co-movement of ER, COPII and cargo pre cargo release (0-2 s before release) further emphasized that both COPII and cargo were linked to the ER and not just closely associated prior to the moment of cargo export.

To visualize the steps of cargo release (Fig. 5C,D; Movies 6 and 7), we analyzed each event prior to cargo moving (0 s), when cargo began to move from the ER but was still connected to ERES signal (1 s) and when cargo had moved from the ERES (2 s). To detect any potential Sec signal over background, a threshold was used to convert Sec24D and Sec31A fluorescent images to binary images (Yen et al., 1995). The cargo images were also converted to binary and this detectible cargo signal set the cargo ROI (yellow outlines, Fig. 5E,F). We then calculated the percentage of Sec24D or Sec31A pixels that were within the cargo ROI at 0, 1 and 2 s for each event (Fig. 5G,H). In the 1 s time points captured, cargo had not yet separated from the ERES, and both Sec24D and Sec31A signals were detected (Fig. 5G,H). To compare Sec24D and Sec31A distribution just as cargo was leaving the ER, we scored the time point at which the cargo ROI was fully separated from the ER and from any residual faint cargo still at the ERES (2 s time point, Fig. 5G-I). At this time point, Sec24D fluorescent signal had little overlap with the cargo ROI (1.2%), whereas Sec31A had a small, yet significantly different (*P*=0.003), percentage overlap with the cargo ROI (12.3%) (Fig. 5I). Thus, a small portion of the outer coat Sec31A protein can remain associated, albeit briefly, with the cargo carrier, but the cargo is released from the ERES in a predominately Sec24D/Sec31A-free cargo carrier. The lack of Sec24D, an inner coat protein, staining suggests that Sec31A plays another role in facilitating cargo export in addition to its role as an outer coat protein. Sec31A has been shown to interact directly with both Sec23 and Sar1, facilitating the GTP hydrolysis of Sar1 and possible subsequent fission of COPII vesicles (Bacia et al., 2011; Bi et al., 2007). Thus, the short lived and asymmetrical distribution of Sec31A (Fig. 5; Fig. S5) on exported cargo domains could be consistent with a model whereby Sec31 functions at the point of fission.

### Rab1 regulates release and trafficking of cargo carriers from ERES

**Fig. 6.**
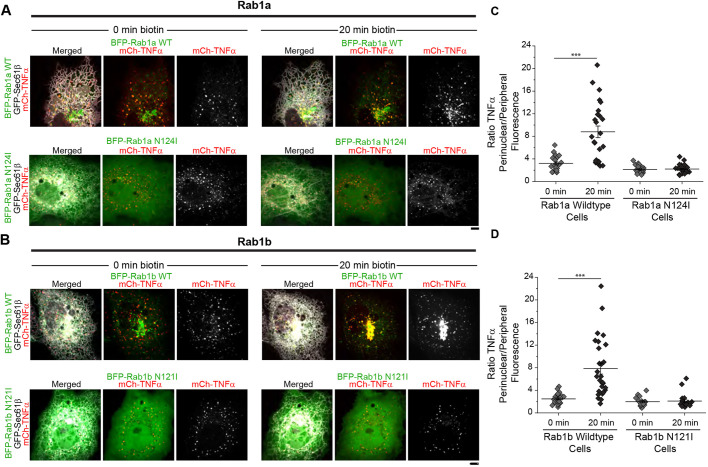
**Rab1 regulates release of cargo carriers from ERES.** (A) Representative examples of cargo localization before or 20 min after biotin treatment in COS-7 cells expressing mCh-TNF RUSH (red), BFP-Rab1a or BFP-Rab1a N124I (green) and GFP-Sec61β (gray). (B) As in A, but for BFP-Rab1b or BFP-Rab1b N121I. (C,D) Quantification of TNF cargo recruitment to the Golgi was measured by tracking fluorescence intensity measured within a 10×10 μm^2^ ROI around the Golgi region (perinuclear area, marked by anti-Giantin) versus peripheral ER at 0 and 20 min following biotin addition in the presence of either BFP-Rab1a, BFP-Rab1a N124I, BFP-Rab1b or BFP-Rab1b-N121I. Redistribution of cargo to the perinuclear/Golgi region was quantified by plotting the ratio of fluorescence intensity of TNF cargo between the perinuclear and peripheral ROIs (***P<0.001). Error bars represent s.e.m. Scale bars: 5 µm.

We also set out to identify membrane markers that would define and traffic with these uncoated cargo carriers. Rab1 is a small GTPase with two mammalian isoforms (Rab1a and Rab1b) that has been shown to regulate the transport of membranes and cargo from the ER to the Golgi (Galea et al., 2015; Martinez et al., 2016; Pind et al., 1994; Plutner et al., 1991; Slavin et al., 2011; Wilson et al., 1994). Rab1 (Ypt1p in yeast) plays an essential role in the secretory pathway, regulating cargo transport from the ER to the Golgi (Bacon et al., 1989; Yuan et al., 2017). Specifically, Rab1 (Ypt1p) has been implicated in regulating COPII vesicle formation and subsequent tethering and fusion of the COPII vesicle at the Golgi membrane (Cai et al., 2008; Cao and Barlowe, 2000; Cao et al., 1998; Morsomme and Riezman, 2002). We thus asked at what steps Rab1 labels and regulates the release and trafficking of uncoated cargo carriers from ERES. As expected, BFP-Rab1a and BFP-Rab1b exogenously expressed in COS-7 cells localized to the cytosol and to the perinuclear/Golgi area within the cell (Fig. 6A) (Plutner et al., 1991; Saraste and Svensson, 1991). BFP-Rab1a and BFP-Rab1b also strongly co-localized with TNFα-SBP-mCh cargo enriched in ERES puncta throughout the ER (Fig. 6). We then used the RUSH system to trap cargo at ERES and asked whether Rab1 regulates cargo release upon biotin addition. Under conditions where we expressed BFP-Rab1, biotin addition resulted in the translocation of TNFα-SBP-mCh cargo to the Golgi (Fig. 6A,B). We generated dominant-negative mutants of Rab1a and Rab1b to test whether trafficking of the mCh-TNF cargo in the RUSH system is regulated by Rab1 family members. Both mutants for Rab1 (Rab1a N124I and Rab1b N121I) are defective in guanine nucleotide binding (Takacs et al., 2017; Wagner et al., 1987; Wilson et al., 1994). The mutant N124I and N121I Rab1 proteins were tolerated at low expression levels in cells, but they did not mark any structures (just cytosolic localization) (Fig. 6A,B). In the presence of dominant-negative forms of Rab1, TNFα-SBP-mCh cargo still accumulated in COPII-marked puncta at 0 min after biotin treatment and the distribution of COPII-marked puncta was unaltered, suggesting that Rab1 is not required for cargo concentration into COPII exit sites (Fig. 6A,B). However, the Rab1 mutants caused a potent block in the export of cargo from the ER, even at 20 min after biotin release (Fig. 6A,B). Cargo redistribution to the perinuclear region of the cell was quantified by tracking the ratio of cargo fluorescence in equally sized ROIs in the perinuclear and peripheral region of the cell. A more positive perinuclear/peripheral ratio marked cells with a higher proportion of cargo signal located in the perinuclear region compared to the peripheral. Significant redistribution of cargo to the perinuclear area of the cell was only observed in cells expressing wild-type Rab1a and Rab1b (*P*<0.001; Fig. 6C,D). SiRNA depletion of Rab1a or Rab1b in HeLa cells similarly impaired trafficking of the Rush cargoes to the Golgi following biotin release compared with controls (*P*<0.0001 for control, *P*=0.04 for Rab1b; Fig. S6). These data confirm that Rab1 family members localize to ERES/cargo carriers and regulate trafficking of these cargo carriers to the Golgi.

### Rab1 labels uncoated cargo carriers

**Fig. 7.**
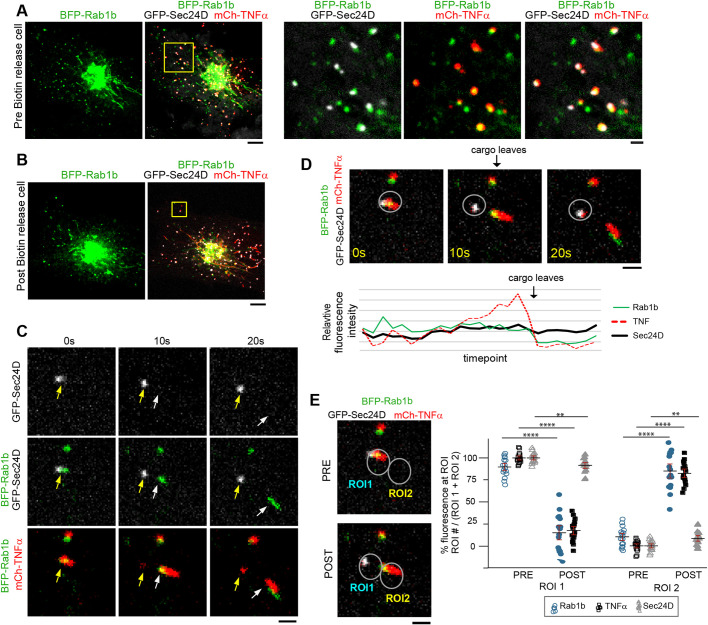
**Rab1 labels uncoated cargo carriers.** (A,B) Representative examples of a COS-7 cell expressing mCh-TNF RUSH (red), GFP-Sec24D (gray) and BFP-Rab1b (green) before (A) or after (B) biotin addition. In A, before biotin addition, the Sec24D, TNF cargo and Rab1b all accumulate in the same place. In B, after biotin is added, Rab1b and cargo localize to structures not marked by Sec24D. (C) Time-lapse image series of an event from B where fluorescently marked cargo (red) and Rab1b (green) are observed leaving the Sec24D-labeled ERES (gray). The original site is marked with a yellow arrow in each frame and the leaving cargo vesicle is marked with a white arrow. (D) For the event in C, BFP-Rab1b, mCh-TNF RUSH cargo and GFP-Sec24D fluorescence intensity was monitored throughout the 2 min movie at the ERES, as indicated by the dotted circle region. Sec24D fluorescence levels do not drop when the cargo leaves. TNF cargo fluorescence and Rab1b fluorescence both drop when cargo leaves (frame defined by the arrow). (E) Quantification of Rab1b, TNF cargo and Sec24D fluorescence immediately before (pre) and immediately after (post) cargo leaves the ERES for multiple export events (16 events from 9 cells). Circles mark where fluorescence measurements were made: ROI1 is the site where cargo leaves from and ROI2 is the site where cargo traffics to. Fluorescence at each region for each marker was background subtracted and the percentage fluorescence at each ROI was calculated pre and post cargo leaving. Rab1b fluorescence follows the cargo fluorescence pre and post cargo leaving (for Rab1 and TNF ****P<0.0001, for Sec24D **P=0.09). Error bars represent s.e.m. Scale bars: 5 µm (A,B), 1 µm (zoomed images in A; C-E).

Finally, we aimed to visualize whether cargo is released from ERES into Rab1 carriers. Given previous reports implicating Rab1b in cargo delivery to the Golgi, we focused on the role of Rab1b in regulating cargo export from ER-associated COPII vesicles. Cos-7 cells were co-transfected with a cargo marker (TNFα-SBP-mCh), a COPII marker (GFP-Sec24D) and fluorescently tagged Rab1b (BFP-Rab1b). Before biotin release, Rab1b signal co-localized with sites where TNFα-SBP-mCh cargo was stalled in Sec24D-marked ERES (Fig. 7A). Upon biotin release, cargo still accumulated at ERES; however, cargo now could be seen in structures marked with Rab1b but not with Sec24D (Fig. 7B,C).

We captured events where TNFα-SBP-mCh cargo disassociated from Sec24D, and in all of these events the carrier was labeled with Rab1b (Fig. 7C-E; Movie 8). Monitoring fluorescence intensity throughout 2 min movies at ERES showed that the Sec24D COPII coat fluorescence remained stable, but there was a drop in intensity for both the Rab1 and TNFα-SBP-mCh fluorescence as cargo left the ERES (Fig. 7D). For these events, we measured fluorescence at the ER exit site (ROI1) and at the site to which cargo was trafficked (ROI2). We analyzed both pre and post cargo leaving frames, and showed that Rab1b followed the cargo signal to ROI2 in the post frame (Fig. 7E). No significant differences between the distribution of Rab1b and cargo signals in the post frames were observed, indicating that when cargo leaves the ERES, it leaves together with Rab1b. By comparison, we saw a significant difference in the distribution of Sec24D in the post frame compared with those of both Rab1b and the cargo (*P*<0.0001 for both cases; Fig. 7E), indicating that the released cargo leaves in a membrane carrier without Sec24D.

Additionally, we measured Rab1b fluorescence distribution in the pre frame and observed a significant difference from that of both cargo (*P*=0.008) and Sec24D (*P*=0.003), suggesting that Rab1b dramatically accumulates at domains marked by Sec24D-cargo prior to export. Given the role of COPII components in regulating the formation and maturation of cargo-loaded vesicles, as well as the role of Rab1 role in Golgi delivery, the delayed accumulation of Rab1b onto cargo-marked structures suggests a potential hand-off between the COPII components and Rab1 at the point of vesicle scission from the ER.

## DISCUSSION

Due to the dynamic nature of cargo trafficking in animal cells, we have optimized a live cell system to visualize co-trafficking of cargo tethered to the ER at exit sites and then subsequent rapid cargo release from the ER in real time. Emerging technology developments have allowed us to use a high frame rate and high-resolution multicolor imaging system with limited phototoxicity to monitor both ERES dynamics on the ER and cargo release from the ER in multiple mammalian cell lines. COPII puncta remain linked to the ER over time and when the ER rearranged along microtubules, these COPII puncta co-trafficked. Interestingly, when cargo left the ER, trafficking away from the ERES, components of the COPII coat remained predominately with the ERES. The COPII pool remained stable on the ER at the ERES, even when cargo was released. These results are consistent with previous work suggesting that ERES are relatively long-lived on the ER (Hammond and Glick, 2000; Shomron et al., 2019 preprint; Stephens et al., 2000). This could indicate an adaptive mechanism by which mammalian cells maximize secretory efficiency by retaining COPII coat proteins at stable ERES. In doing so, it ensures that COPII fulfills the primary role of partitioning secretory cargo, providing a quality control checkpoint that selectively advances the forward transport of properly folded cargo (Mezzacasa and Helenius, 2002). Given the expansive network of the ER in mammalian cells and the sheer volume of the cytoplasm it might be advantageous to maintain COPII organization on the ER to provide stable sites where proteins can accumulate. Taken together with our data, it is intriguing to postulate that the stable organization of COPII proteins on the ER provides specificity and regulatory control over the way cargo is compartmentalized and organized prior to export.

We observed that cargo and COPII markers moved together with the ER on the microtubules but when released, cargo left the ER in COPII-free puncta. This suggests that the cargo is being trafficked away from the ER in a vesicle or cluster of vesicles, largely unmarked by COPII. We asked, if not COPII, what could be regulating this trafficking? We specifically looked for other membrane components thought to regulate ER to Golgi trafficking. In a report by Slavin et al. (2011), inhibition of Rab1b activity was sufficient to delay cargo sorting at the ER. We found that both Rab1a and Rab1b localized to cargo-containing COPII-labeled sites still attached to the ER before tracking with the COPII coat-free cargo carriers upon export from the ER. This, together with our data, suggests that Rab1 is a necessary regulator of the unmarked cargo vesicle that releases from COPII-marked sites before export to the Golgi.

Our observation that Rab1b is recruited to COPII-marked sites prior to cargo export suggests that Rab1b recruitment is an early event in secretory trafficking that promotes cargo export from COPII-coated domains on the ER. We additionally report that when a dominant-negative Rab1 is introduced or when Rab1 is depleted, cargo cannot accumulate at the Golgi region and remains at ERES puncta. Wild-type Rab1a and Rab1b both localize to ERES, but the dominant-negative forms of Rab1 localize diffusely in the cytoplasm and do not localize to the cargo puncta in the ER that is stuck at ERES. These findings further emphasize the importance of Rab1 at the early steps of cargo release from ERES. However, Rab1 could be playing this upstream role at ERES and still play a downstream role to aid in later fusion with the Golgi. In yeast, Rab1 regulates the tethering of COPII vesicles to Golgi acceptor membranes and may therefore play a similar role in mammalian cells (Barlowe, 1997; Nakajima et al., 1991). Rab1b continues to track with uncoated carriers and may therefore function to facilitate eventual fusion with the Golgi, potentially through the subsequent recruitment of COPI machinery.

Emerging technological developments have allowed us to use higher frame rate imaging and a more exhaustive examination of the dynamics of cargo export from the ER in mammalian cells. We observed cargo leaving ERES in a carrier unmarked by COPII proteins. Importantly, Rab1 localized to the cargo prior to export from the ER and tracked with cargo as it trafficked away. Due to the dynamic nature of cargo trafficking, a live cell system was needed to understand how ER to Golgi trafficking occurs in real time. This study monitored the role of several major ER cargo trafficking players and visually constructed the stepwise hand-offs required for cargo movement from the ERES to the microtubule trafficking roadways. This visual framework opens up future studies for probing how each of these pathway components is regulated and how this stepwise process can be altered or potentially rerouted following various perturbations to cellular homeostasis.

## MATERIALS AND METHODS

### Tissue culture

COS-7 (ATCC-CRL-1651), HeLa (ATCC-CCL-2) and U2OS (ATCC-HTB-96) cells were purchased from ATCC (Table S1). COS-7 and HeLa cells were grown in Dulbecco's modified Eagle's medium (DMEM) supplemented with 10% fetal bovine serum (FBS) and 1% penicillin/streptomycin. U2OS cells were grown in McCoy's 5A medium supplemented with 10% FBS and 1% penicillin/streptomycin.

### DNA plasmids

Steptavidin (Str)-KDEL-TNFα-SBP-mCh, Str-KDEL-ManII-SBP-EGFP, GFP-Sec16s, GFP-Sec23A, GFP-Sec24D, GFP-Sec31A and EGFP-VSVG were acquired from Addgene (Table S1) (Bhattacharyya and Glick, 2007; Boncompain et al., 2012; Presley et al., 1997; Richards et al., 2011; Stephens et al., 2000). The mCh-KDEL, BFP-KDEL and mCh-tubulin were previously described (Friedman et al., 2010, 2011; Zurek et al., 2011). Ras-related protein Rab1b (NM_030981.3) was cloned from HeLa cDNA and inserted into *Xho*1/*Kpn*1 sites of the pAcGFP-C1 (Clontech, Mountain View, CA) to make GFP-Rab1b. Site-directed mutagenesis was used to generate the dominant-negative GFP-Rab1b N121I, which is unable to bind nucleotides (Takacs et al., 2017). BFP-Rab1b wild-type (WT) and BFP-Rab1b N121I were generated by subcloning Rab1b WT and Rab1b N121I and inserting them into *Xho*1/*Kpn*1 sites of mTag-BFP (Evrogen, Russia).

### Transfection and imaging

Prior to imaging experiments, all three cell types were seeded in six-well, plastic-bottom dishes at 1×10^5^ cells/ml about 18 h prior to transfection. Plasmid transfections were performed as described previously (Hoyer et al., 2018). The following standard amounts of DNA were transfected per ml: 0.1 µg GFP-Sec24D, 0.1 µg YFP-Sec31A, 0.1 µg GFP-Sec23A, 0.1 µg GFP-Sec16s, 0.2 µg BFP-KDEL, 0.2 µg mCh-KDEL, 0.075 µg mCh-tubulin, 0.4 µg Str-KDEL-TNFα-SBP-mCh, and Str-KDEL-ManII-SBP-EGFP, 0.2 µg EGFP-VSVG and 0.075 µg Rab1a or Rab1b (WT or mutant) fluorescent constructs.

All images (except those used for FRAP analysis) were acquired on an inverted fluorescence microscope (TE2000-U; Nikon) equipped with a Yokogawa spinning-disk confocal system (CSU-Xm2; Nikon or Yokogawa CSU X1). Images were taken with a 100× NA 1.4 oil objective on an electron-multiplying charge-coupled device (CCD) camera 50×50 (Cascade II; Photometrics), 50×50 (Andor) or 1TB (Andor). Lasers were aligned to release at least 12 Mw out of the optic cable prior to experiments. Images were acquired with Nikon Elements or with Micromanager and then analyzed, merged and contrasted using Fiji (ImageJ), as well as converted to 400 dpi using Photoshop (Adobe, San Jose, CA). Scale bars were generated using Fiji (Schindelin et al., 2012, https://fiji.sc/). Movies 1-8 were generated using ImageJ, Adobe Photoshop and Quicktime. Live cells were imaged at 37°C in prewarmed Fluorobrite supplemented with 10% FBS. In ionomycin experiments, COS-7 cells were imaged just prior and 5 min after ionomycin addition (2 µM). For all immunofluorescence experiments, cells were first fixed at room temperature with 4% paraformaldehyde plus 0.5% glutaraldehyde in PBS, solubilized in 0.1% Triton-X in PBS and then immunostained. Anti-Giantin was used at 1:500 and AlexaFluor 647-conjugated antibody (Thermofisher) was used at 1:300. Images of fixed cells were captured at room temperature.

Fluorescence recovery assays (FRAP) were performed at the University of Colorado BioFrontiers Advanced Light Microscopy Core on a Nikon A1R Laser Scanning Confocal equipped with an Andor Ixon 3 EMCCD camera (DU897E-C50). ROIs of 10×10 µm were bleached with a 488 nm laser to selectively target the GFP-marked exit sites in the peripheral ER. Images were captured with a 100× NA 1.45 oil objective. Cells were tracked for 2 min after photobleaching to monitor fluorescence recovery.

### Image analysis

All image processing was performed using Fiji (Schindelin et al., 2012). To track association of exit sites with the peripheral ER, 10 µm ROIs were used to follow the movement of exit sites throughout a 2 min movie; frames were taken every 5 s. Only exit sites that remained associated with the ER throughout the extent of the entire movie were marked as having remained associated with the ER. Exit sites that moved out of the focal plane or became detached for more than two consecutive frames were marked as not associated. To track exit site dynamics, exit site trajectories were tracked by drawing a segmented line connecting the location of a particular exit site throughout a 2 min movie. Total distance (in microns) was calculated by summing the entire distance traveled. Maximum velocity was the speed calculated by dividing the maximum distance traveled between adjacent images in the time lapse by 5 s (images acquired every 5 s). Fluorescence recovery was measured by calculating the change in fluorescence immediately after photobleaching and 30 s later, compared with the measured fluorescence of the exit site immediately prior to photobleaching. All *t*-tests performed assumed unequal variance.

### Cargo export analysis

The following protocol was modified from that first described by Boncompain et al. (2012) to track the export dynamics of RUSH cargo. Briefly, COS-7 cells were seeded in six-well dishes and transfected with the following 3× fluorescently tagged constructs: (1) ER marker, (2) exit site marker and (3) secretory RUSH cargo marker according to the protocol described above. At 24 h after transfection, medium was replaced with prewarmed imaging medium (Fluorobrite plus 10% FBS). COS-7 cells were marked for imaging and z-stacks that went throughout the entire cell were acquired just prior to biotin addition to initiate cargo release from the ER. Biotin was added to a final concentration of 40 µM and z-stacks of marked cells were taken at the time points indicated.

To quantify cargo recruitment to the ER, one 5×5 μm^2^ area was selected in the peripheral ER and one 5×5 μm^2^ area was drawn around the Golgi (marked by Giantin). The ratio of cargo fluorescence for each cell at 0 min and 20 min after cargo release following addition of biotin was used as a proxy to measure cargo accumulation at the Golgi.

Live cell images tracking cells every 5 s after biotin addition were also acquired to track the dynamics of cargo over time following release from the ER. When cargo moved away from the site marked with coat towards the perinuclear area of the cell, the frames pre and post cargo movement were analyzed. Fluorescent measurements were made at both ROI1 (where the cargo initially rested) and ROI2 (where the cargo moves to) in the pre and post frames. The ROIs were the same size and each ROI measurement was background subtracted. Percentage fluorescence at each ROI1 was calculated by comparing fluorescence in one ROI to total fluorescence in both ROI1 and ROI2 in either the pre or post frames [ROI#/ (ROI1+ROI2)] The same analysis was performed with cells transfected with exit site marker, cargo and BFP-Rab1 to monitor Rab1 moving away with the cargo signal.

VSVG cargo export dynamics were monitored in real time following the modified protocol from Presley et al. (1997). COS-7 cells were seeded in a six-well dish and transfected with the following 3× fluorescently tagged constructs: (1) ER marker, (2) exit site marker and (3) VSVG cargo marker according to the protocol described above. At 5 h after transfection, medium was replaced with prewarmed imaging medium (Fluorobrite plus 10% FBS) and cells were stored at 40°C overnight. The following morning (∼18 h after transfection), COS-7 cells were moved to a live cell chamber equilibrated at 32°C. Individual cells were allowed to equilibrate for 5 min at the new temperature before being tracked every 2 s for 60 s. Cargo dynamics were analyzed as described above.

For Sec24D and Sec31A signal tracking with cargo at a high frame rate (Fig. 6), 5×5 µm cropped images of the ERES with cargo release event were thresholded using Yen threshold in Fiji image analysis software to convert the fluorescent images to binary (Yen et al., 1995). With the cargo binary images, the analyzed particles function was used to create the cargo ROI. Then, with the SEc24D or Sec31A binary images, the percentage of positive pixels in the cargo ROI was calculated and this was termed percentage coverage. The number of cells analyzed is indicated and *P* was calculated using Student's *t*-tests assuming unequal variance.

### Rab1a/Rab1b knockdown

RNAi knockdown of Rab1a and Rab1b was performed using ON-TARGETplus Human siRNASMARTpools (Horizon Discovery). HeLa cells were seeded at 150,000 cells per well of a six-well dish, ∼16 h prior to transfection. Cells were first transfected ∼48 h before fixation with 5 µl Dharmafect (Horizon Discovery) in Opti-MEM medium with 25 nM of siRNA SMARTpool or 25 nM Silencer Negative Control #1 siRNA (Ambion AM4635). After 5 h of transfection, cells were washed and replenished with DMEM medium supplemented with 10% FBS and 1% penicillin/streptomycin. After 24 h, cells were transfected again with plasmid DNA as described above (0.4 µg Str-KDEL-TNFα-SBP-mCh, 0.2 µg BFP-KDEL, 0.1 µg GFP-Sec24D) with the addition of 12.5 nM siRNA SMARTpool or Silencer Negative Control. Cells were seeded in 35 mm imaging dishes (CellVis) and analysis of cargo export was performed at 48 h after knockdown as described above.

HeLa cell lysates were collected at 48 h after knockdown and protein knockdown was confirmed by SDS-Page as described previously (Hoyer et al., 2018). Blots were incubated overnight with primary antibodies against Rab1a (CST #13075) and Rab1b (Thermo #PA5-68302) at 1:1000 dilution. α-Tubulin (Sigma T3562) was used at 1:5000 as loading control. HRP-conjugated goat anti-rabbit antibody (Sigma-Aldrich) was used at 1:1000 as described previously (Hoyer et al., 2018). Signal was detected with SuperSignal Femto Chemiluminescent Substrate (Thermo).

## Supplementary Material

Supplementary information

Reviewer comments
