# Supplementary material for: Vesicular and uncoated Rab1-dependent cargo carriers facilitate ER to Golgi transport
Source: J Cell Sci. 2020 Jul 24;133(14):jcs239814. doi: 10.1242/jcs.239814 (PMC7390636; doi:10.1242/jcs.239814)
Supplement: Supplementary information [file joces-133-239814-s1.pdf]

Table S1: Key Resources

**Reagent type (species or resource)**	**Designation**	**Source or Reference**	**Identifiers**	**Additional Information**
Cell Line (*Cercopithecus aethiops*)	COS-7	ATCC	ATCC-CRL-1651	DMEM + 10% FBS + 1% P/S
Cell Line (*Homo sapiens*)	HeLa	ATCC	ATCC-CCL-2	DMEM 10% FBS + 1% P/S
Cell Line (*Homo sapiens*)	U2OS	ATCC	ATCC-HTB-96	McCoy's 5A + 10% FBS + 1% P/S
Antibody	Anti-Giantin (rabbit)	BioLegend	Biolegend cat# 924302	1:500
Antibody	Alexa_Fluor 647 (anti-rabbit)	ThermoFisher	Thermofisher A-212245	1:300
Plasmid	pEGFP-Sec23A	Addgene	Addgene: 66609	pEGFP-Sec23A was a gift from David Stephens
Plasmid	pEGFP-Sec24D	Addgene	Addgene: 32678	pEGFP-Sec24D was a gift from Henry Lester
Plasmid	pmGFP-Sec16s	Addgene	Addgene: 15775	pmGFP-Sec16S was a gift from Benjamin Glick
Plasmid	pEYFP-Sec31A	Addgene	Addgene: 66613	pEYFP-Sec31A was a gift from David Stephens
Plasmid	Str_KDEL_TNFalpha_SBP_EGFP	Addgene	Addgene: 65278	Str_KDEL_TNFalpha RUSH was a gift from Franck Perez
Plasmid	Str_KDEL_TNFalpha_SBP_mcherry	Addgene	Addgene: 65279	Str_KDEL_TNFalpha RUSH was a gift from Franck Perez
Plasmid	Str_KDEL_ManII_SBP_EGFP	Addgene	Addgene: 65252	Str_KDEL_ManII RUSH was a gift from Franck Perez
Plasmid	pEGFP_VSVG	Addgene	Addgene: 11912	pEGFP-VSVG was a gift from Jennifer Lippincott-Schwartz
Plasmid	mcherry_tubulin	Friedman et al, JCB 2010		
Plasmid	GFP_Rab1b WT	HeLa cDNA		
Plasmid	GFP_Rab1b_N121I	HeLa cDNA		

Journal of Cell Science • Supplementary information

Plasmid	mNeon-Sec24D			mNeon subcloned in to pEGFP-Sec24D to replace EGFP
Plasmid	mNeon-Sec31A			mNeon subcloned in to pEYFP-Sec21A to replace YFP
Chemical	Biotin	Sigma	Sigma: B4501	Re-suspended in PBS
Chemical	Ionomycin	Invitrogen	I24222	2 μM

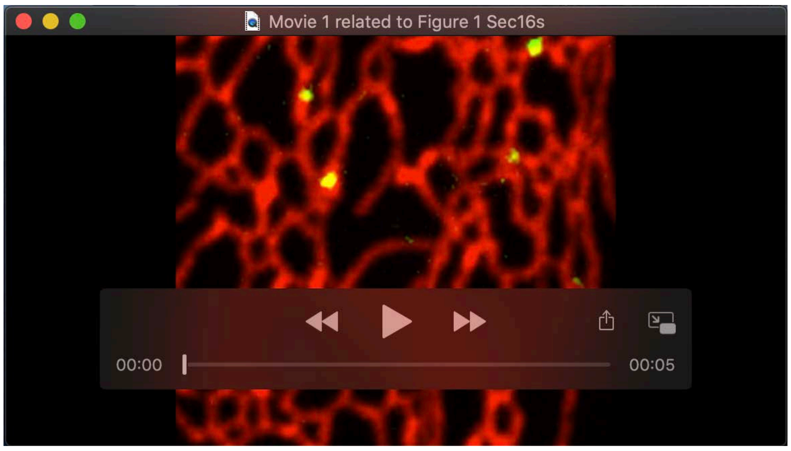

Movie 1, related to Figure 1. Sec16s puncta localize to stable domains on peripheral ER tubules. 10x10 micron region of a COS-7 cell expressing mch-KDEL (ER in red) and GFP-Sec16s (COPII component in green), imaged for 2 minutes with 5 second intervals.

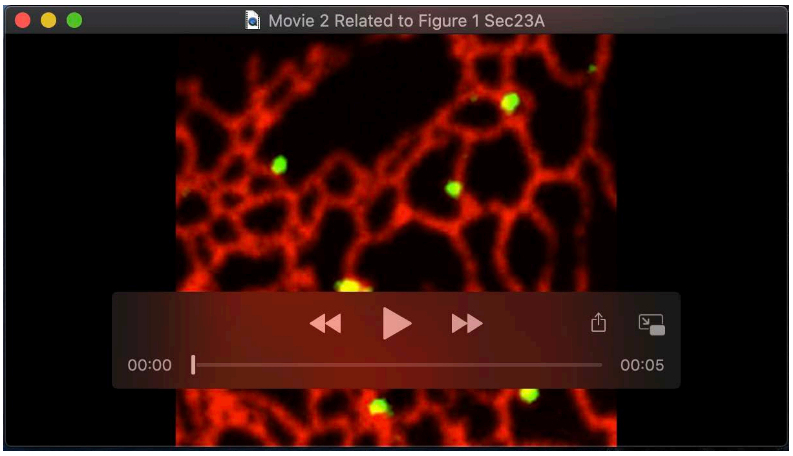

Movie 2, related to Figure 1. Sec23A puncta localize to stable domains on peripheral ER tubules. 10x10 micron region of a COS-7 cell expressing mch-KDEL (ER in red) and GFP-Sec23A (COPII component in green), imaged for 2 minutes with 5 second intervals.

Journal of Cell Science • Supplementary information

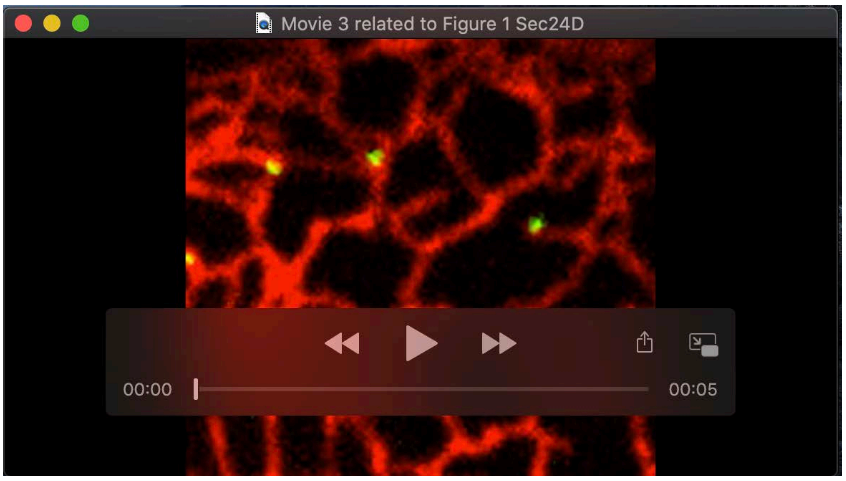

Movie 3, related to Figure 1. Sec24D puncta localize to stable domains on peripheral ER tubules. 10x10 micron region of a COS-7 cell expressing mch-KDEL (ER in red) and GFP-Sec24D (COPII component in green), imaged for 2 minutes with 5 second intervals.

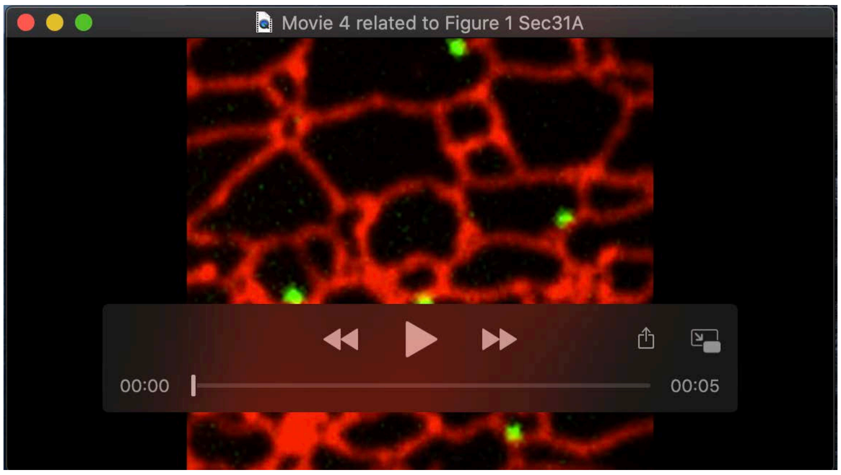

Movie 4, related to Figure 1. Sec31A puncta localize to stable domains on peripheral ER tubules. 10x10 micron region of a COS-7 cell expressing mch-KDEL (ER in red) and GFP-Sec31A (COPII component in green), imaged for 2 minutes with 5 second intervals.

Journal of Cell Science • Supplementary information

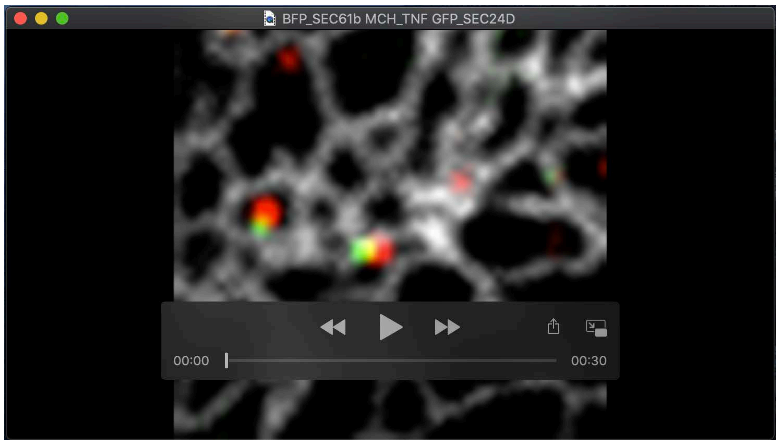

Movie 5, related to Figure 5. COPII components remain linked to the ER as cargo is exported. 5x5 micron region of a COS-7 cell expressing BFP-Sec61β (ER in gray), mCh-TNF RUSH (cargo in red), and GFP-Sec24D (COPII component in green), imaged for 2 minutes with 5 second intervals. This movie shows a 50 sec time period (10 frames of the image sequence) highlighting when cargo moves from two ER-linked COPII sites. The yellow arrow marks the first cargo export event and the yellow arrowhead marks the second cargo export event.

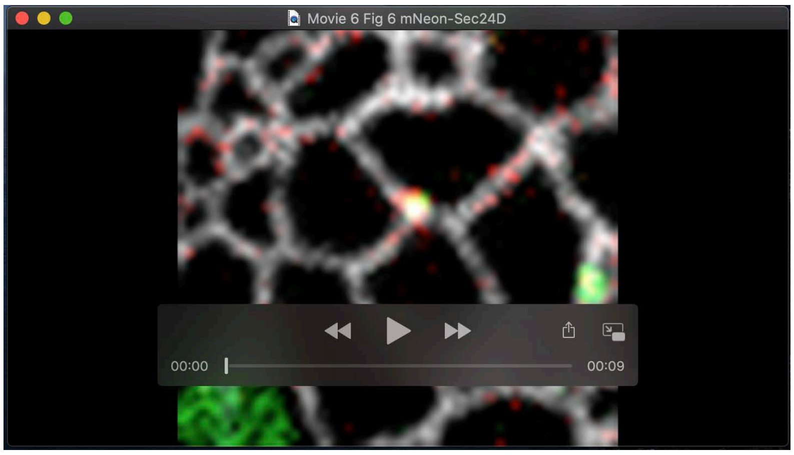

Movie 6, related to Figure 6. Rapid time-lapse imaging of release from Sec24D labelled ERES. 5x5 micron region of a COS-7 cell expressing BFP-Sec61β (ER in gray), mCh-TNF RUSH (cargo in red), and GFP-Sec24D (COPII component in green), imaged for 2 minutes with 1 second intervals. This movie shows a 5 sec time period (5 frames of the image sequence) highlighting when cargo moves from the ER-linked Sec24D site.

Journal of Cell Science • Supplementary information

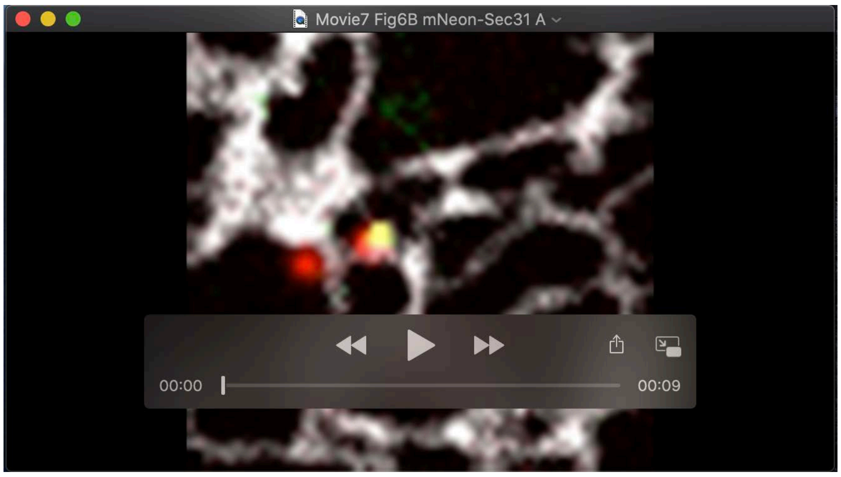

Movie 7, related to Figure 6. Rapid time-lapse imaging of release from Sec31A labelled ERES. 5x5 micron region of a COS-7 cell expressing BFP-Sec61β (ER in gray), mCh-TNF RUSH (cargo in red), and GFP-Sec31A (COPII component in green), imaged for 2 minutes with 1 second intervals. This movie shows a 10 sec time period (10 frames of the image sequence) highlighting when cargo moves from the ER-linked Sec31A site and when Sec31A signal diminishes.

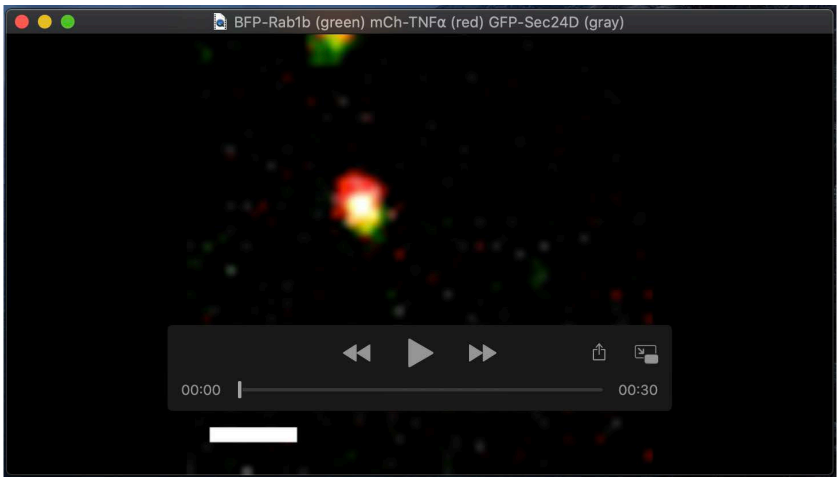

Movie 8, related to Figure 8. Figure 8: Rab1 labels uncoated cargo carriers. 5x5 micron region of a COS-7 cell expressing BFP-Rab1b (Rab1b in green), mCh-TNF RUSH (cargo in red), and GFP-Sec24D (COPII component in gray), imaged for 2 minutes with 5 second intervals. This movie shows a 50 sec time period (10 frames of the image sequence) highlighting when cargo moves from the ER-linked Sec24D site in a Rab1b labelled vesicle.

Journal of Cell Science • Supplementary information

Figure S1

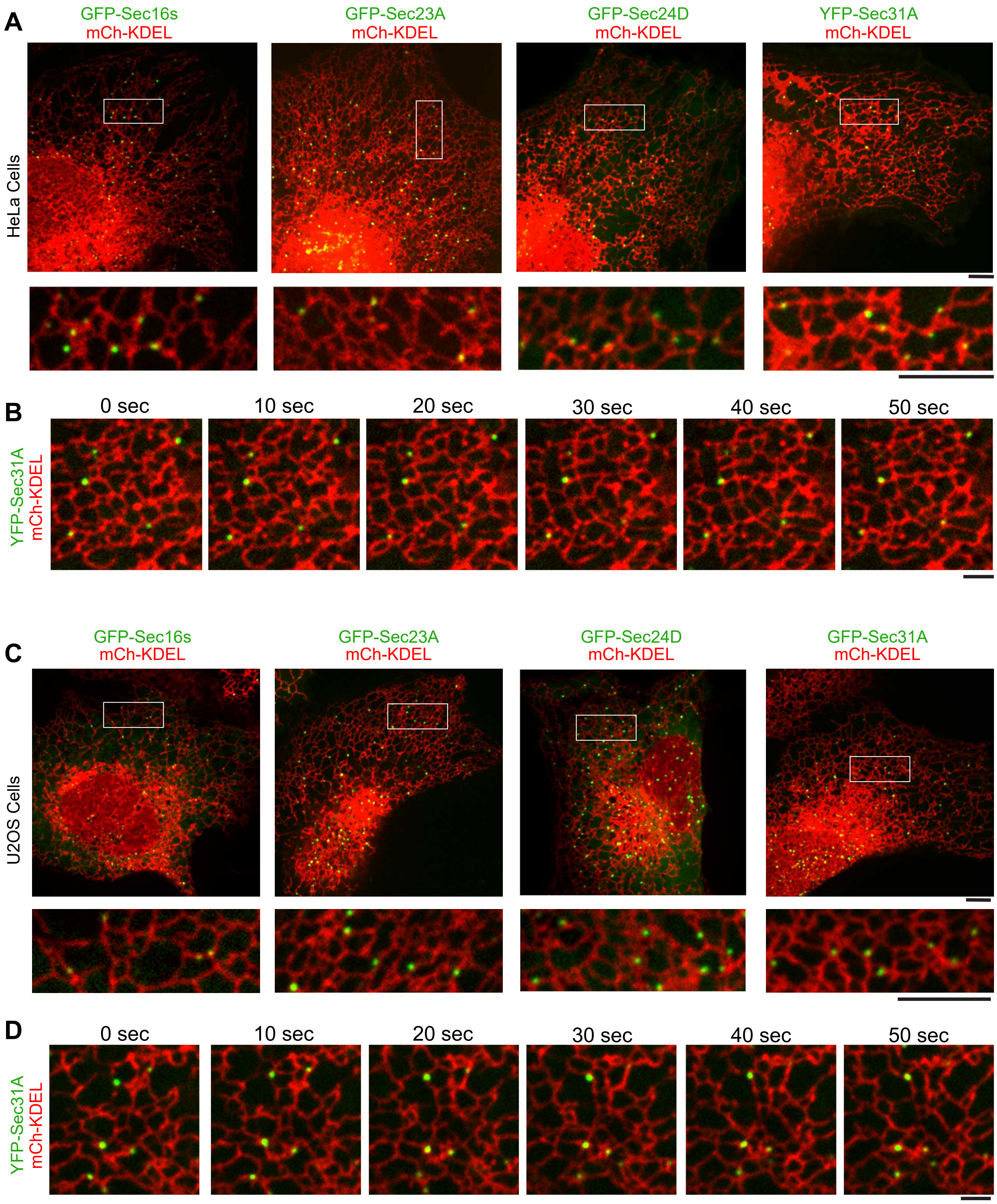

Figure S1: COPII Exit Sites are Tightly Associated with the Peripheral ER in HeLa and U2OS
A and C. Distribution of ERES within the peripheral ER (mch-KDEL, red) of HeLa (A) or U2OS (C) cells expressing fluorescently tagged markers of COPII exit sites (GFP-Sec16s, GFP-Sec23A, GFP-Sec24D, and YFP-Sec31A, green). Scale bars 5 µm. B and D. Exit Sites (YFP-Sec31A, green) were tracked over time for 2 minutes to quantify whether sites were associated with ER (mch-KDEL, red) throughout the 2 minutes. Scale bars 2 µm.

Journal of Cell Science • Supplementary information

Figure S2

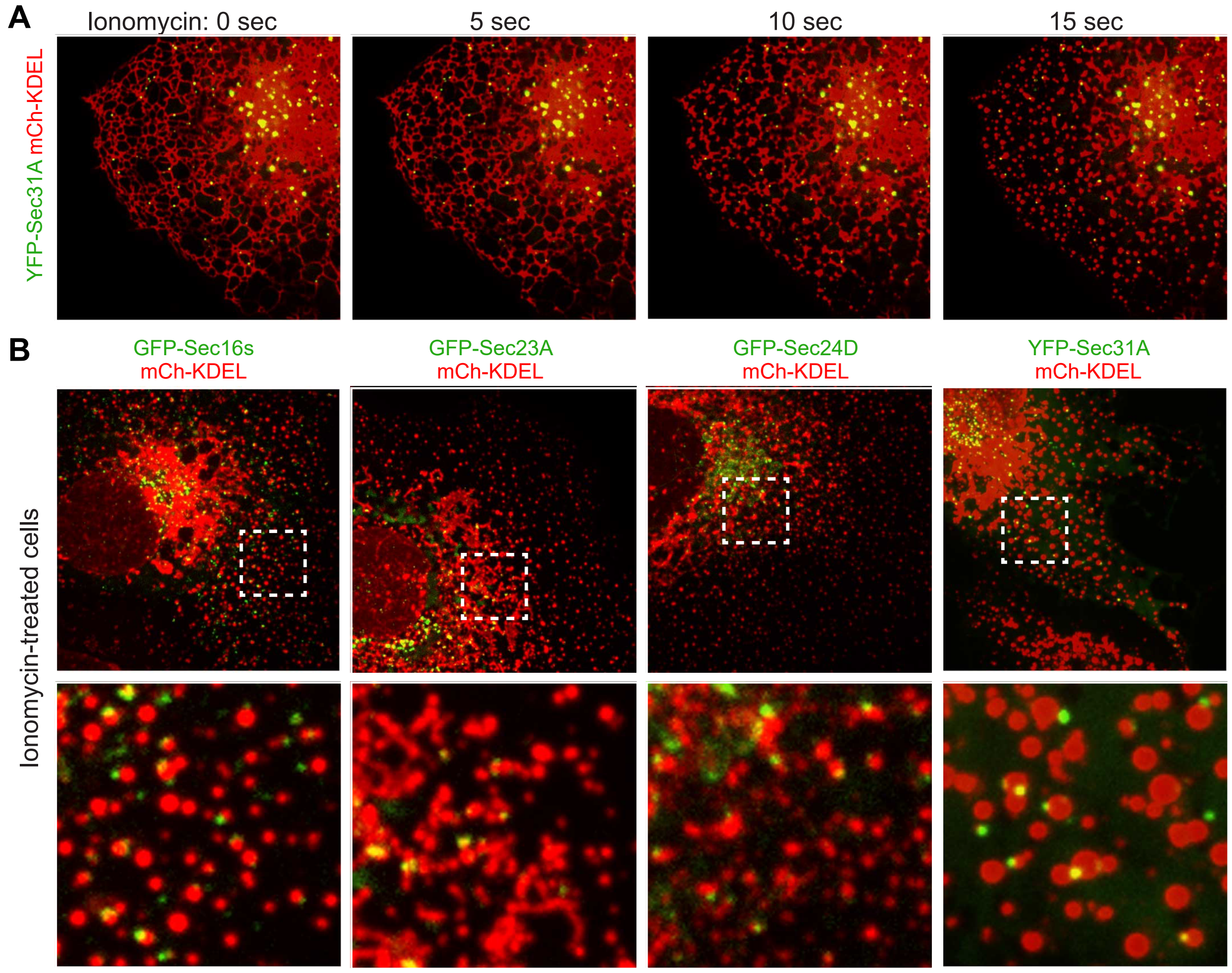

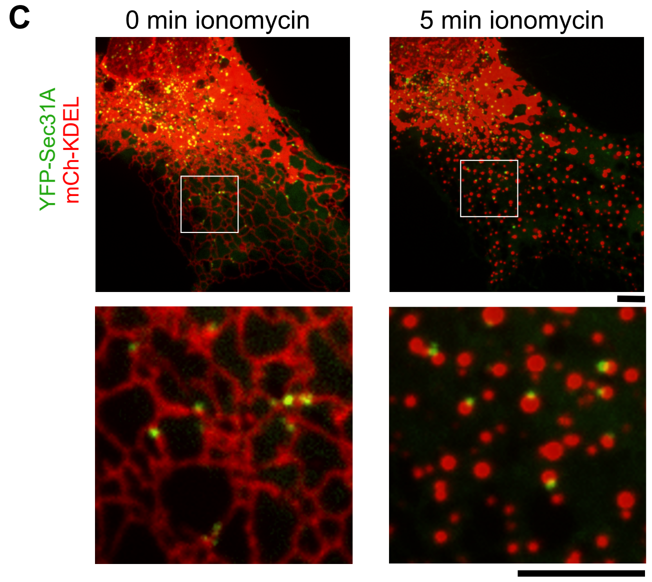

D

Ionomycin treated COS-7 cells

COPII marker	# of ERES	% associated
Sec16s	348	86.7 ± 13.6 %
Sec23A	293	91.3 ± 10.2 %
Sec24D	113	94.1 ± 6.8 %
Sec31A	259	89.3 ± 15.3 %

Figure S2: COPII components remain ER-associated upon ionomycin-induced fragmentation. A. Representative images of COS-7 cells expressing fluorescently tagged markers of the ER (mch-KDEL, red) and COPII exit sites (YFP-Sec31A, green) before and after (2 μM) ionomycin treatment. Zoomed insets (from white square in whole cell view) show exit site association with ER following ionomycin fragmentation. B. Quantification of the percentage of ER exit sites associated with the ER following ionomycin treatment. N = 3 replicates; 20 cells for Sec16s, 20 cells for Sec23A, 11 cells for Sec24D, and 23 cells for Sec31A. Scale Bars: 5 μm for both whole cell and zoomed insets.

Journal of Cell Science • Supplementary information

Figure S3

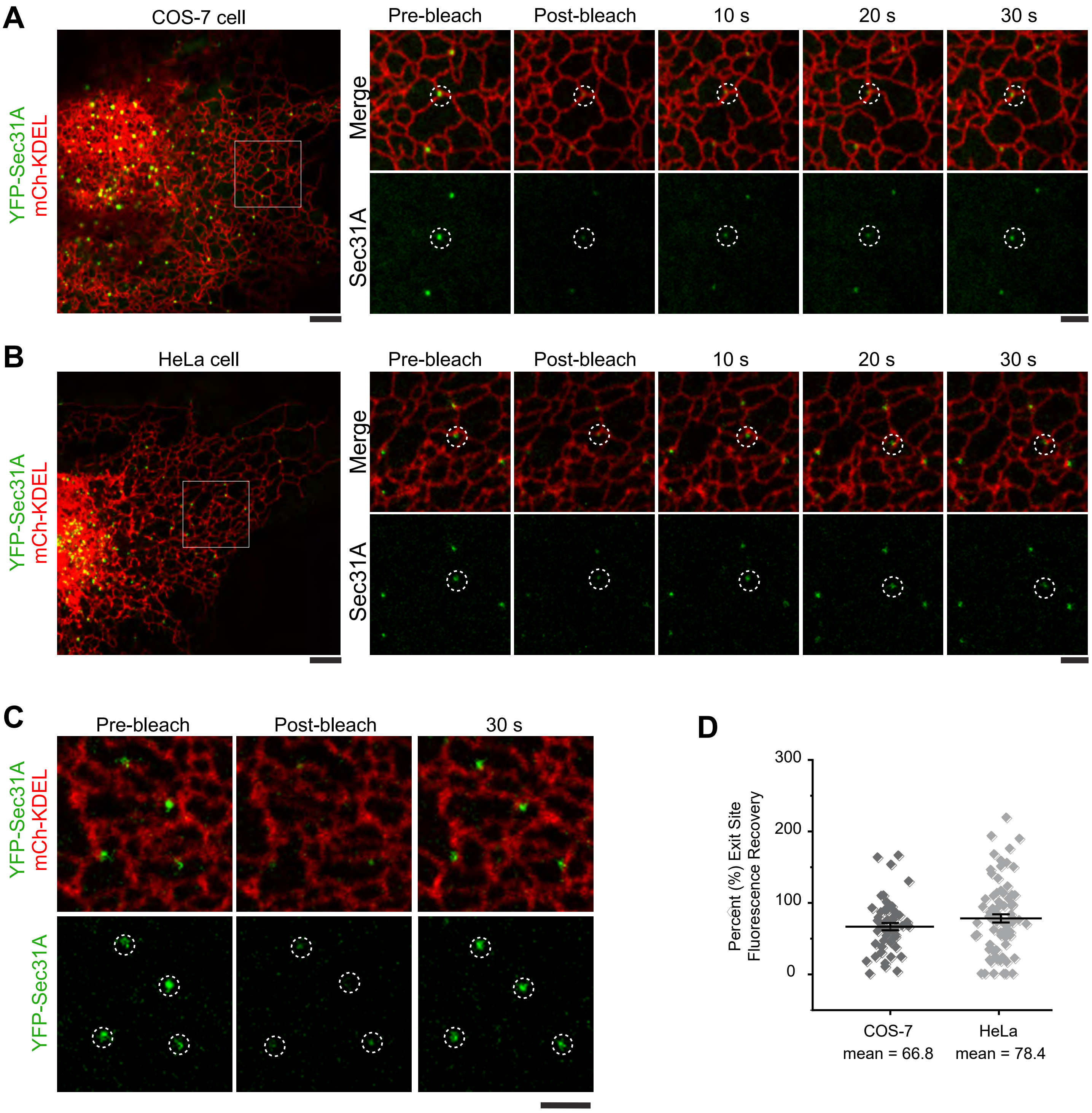

Figure S3: COPII coat proteins cycle at long lived ERES.
A. A COS-7 cell expressing fluorescent markers to label the ER network (mCh-KDEL) and YFP-Sec31A (in green). Zoomed insets (from white square in whole cell view) show the temporal dynamics of Sec31A fluorescence before and after targeted photobleaching within the ROI indicated. White circles track the fluorescence of an individual COPII puncta during recovery. B. As described in A but for a HeLa cell. C. Representative images demonstrating the quantification protocol used to measure fluorescence recovery by measuring fluorescence intensity within the dashed circles at pre, post and 30 seconds after photobleaching. D. Quantification of fluorescence recovery (at 30 sec relative to fluorescence just prior to photobleaching) for COS-7 and HeLa cells (from 60 and 78 events in 13 COS7 and 14 HeLa cells, respectively). Error bars represent standard error about the mean. A-C scale bars = 5 µm for the whole cell and 2µm for zoom insets.

Journal of Cell Science • Supplementary information

Figure S4

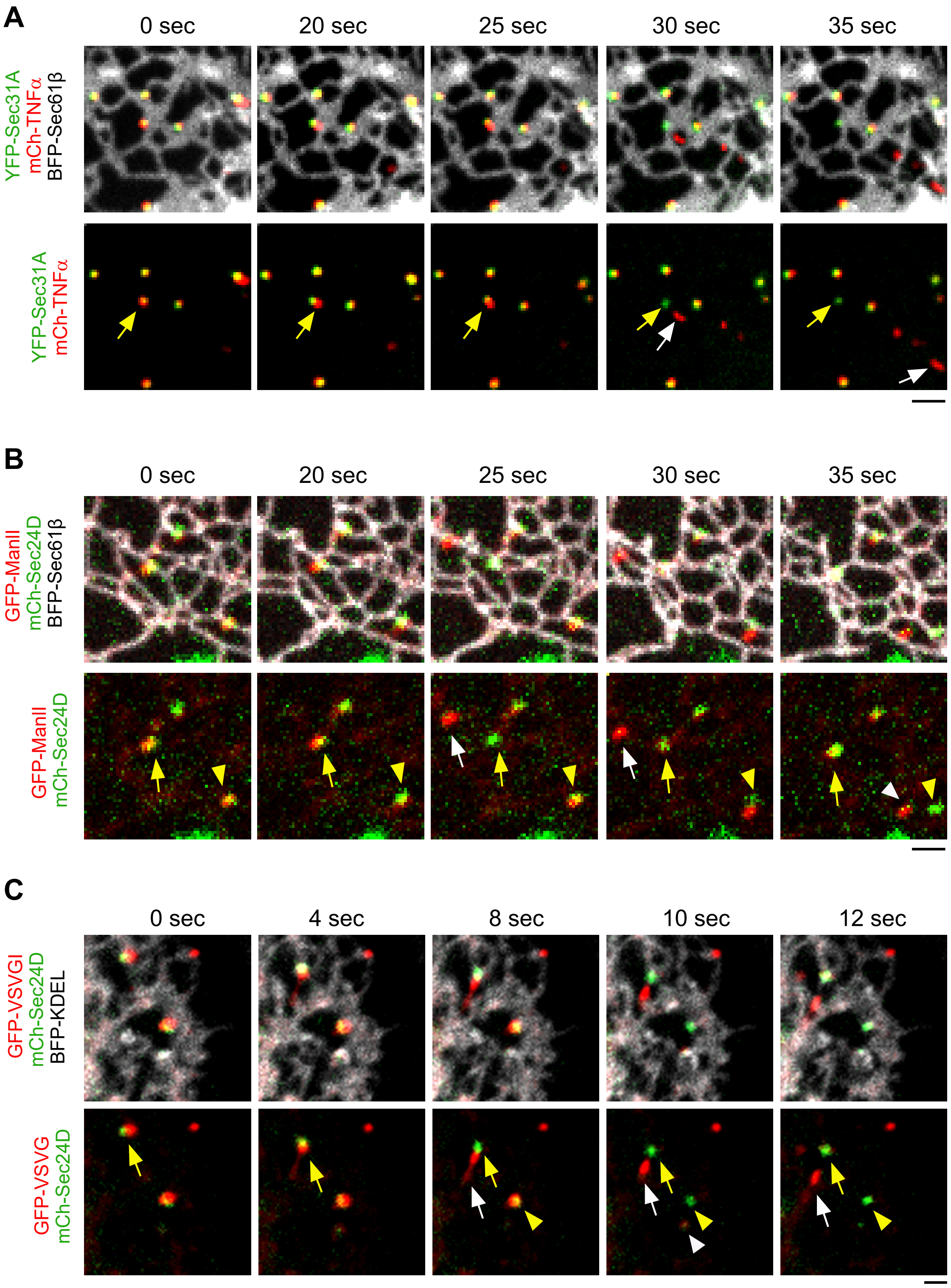

Figure S4: COPII ERES remain linked to the ER with various coat or cargo markers
A. Example time-lapse image series of events in COS-7 cells where fluorescently marked cargo (mCh-TNF,red) is observed leaving the COPII fluorescent puncta (YFP-Sec31A, green) on the ER (BFP- Sec61β, gray) after biotin addition. B As in A for a different cargo (GFP-ManII RUSH, red), a different coat (mCh-Sec24D, ER (BFP- Sec61β, gray). C. As in B but for a different cargo (GFP-VSVG), ER (BFP-KDEL). Arrow marks the first event and if there is a second event, the arrowhead marks the second event. The ERES is marked with yellow in each frame and the leaving cargo vesicle is marked in white. Scale bars 1 µm.

Figure S5

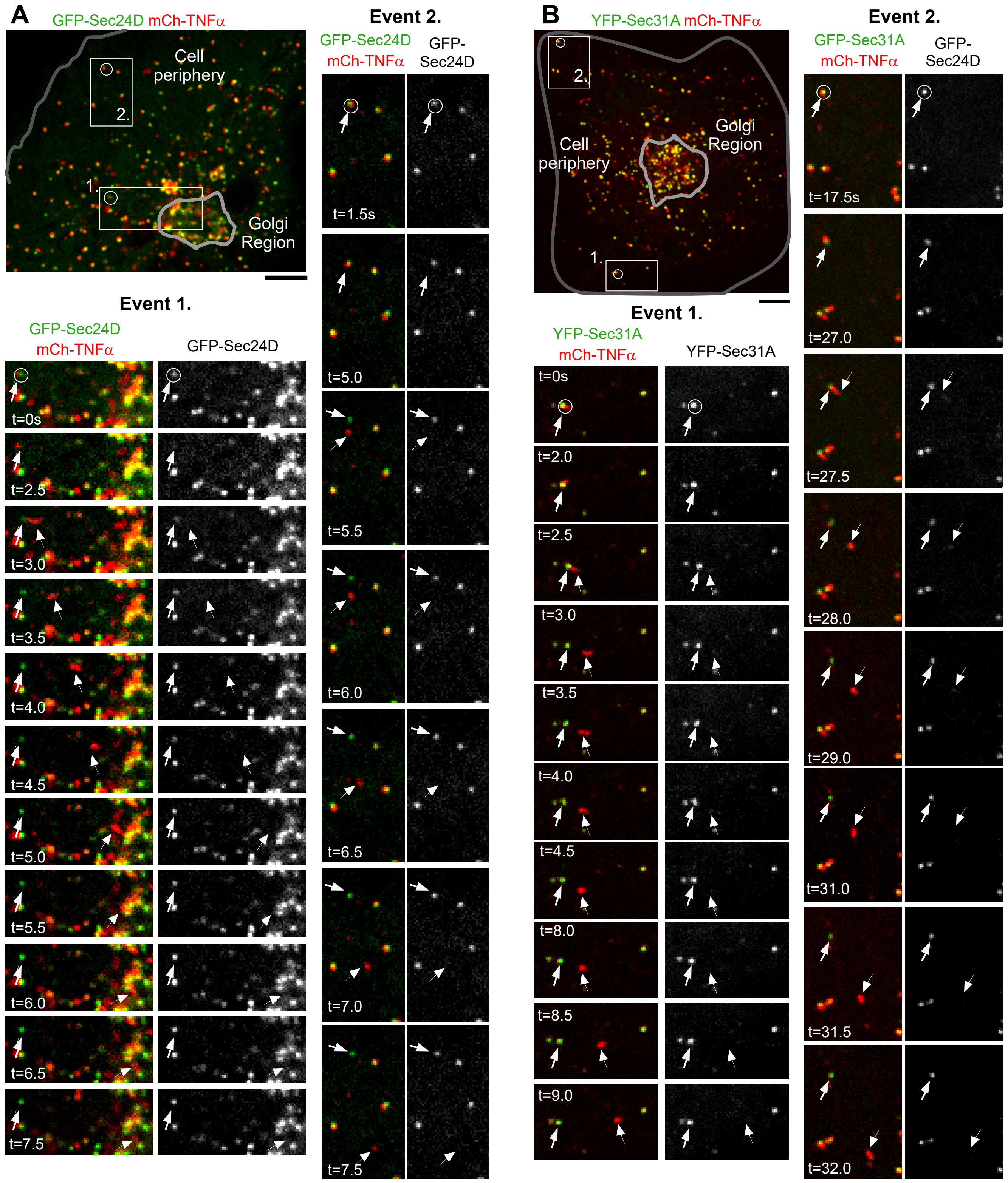

Figure S5. Rapid time-lapse imaging of release suggests cargo is extruded not uncoated. A. A COS-7 cell expressing mCh-TNF RUSH (red) and GFP-Sec24D (green) with marked insets labeling two different events where fluorescently marked cargo (red) is observed leaving COPII fluorescent puncta (green) on the ER and tracked through time. In the zoomed insets, white circles mark the beginning position of the dual labeled Sec24D and TNFalpha marked site and white arrows track the dynamics of the TNFalpha cargo (red) over time. GFP-Sec24D only panels demonstrate dynamics of COPII coat which does not track with cargo (arrows). B. As in A for mCh-TNF RUSH cargo (red) and YFP-Sec31A (green). The Golgi region is highlighted in grey. Scale bar 5 μm

Journal of Cell Science • Supplementary information

Figure S6

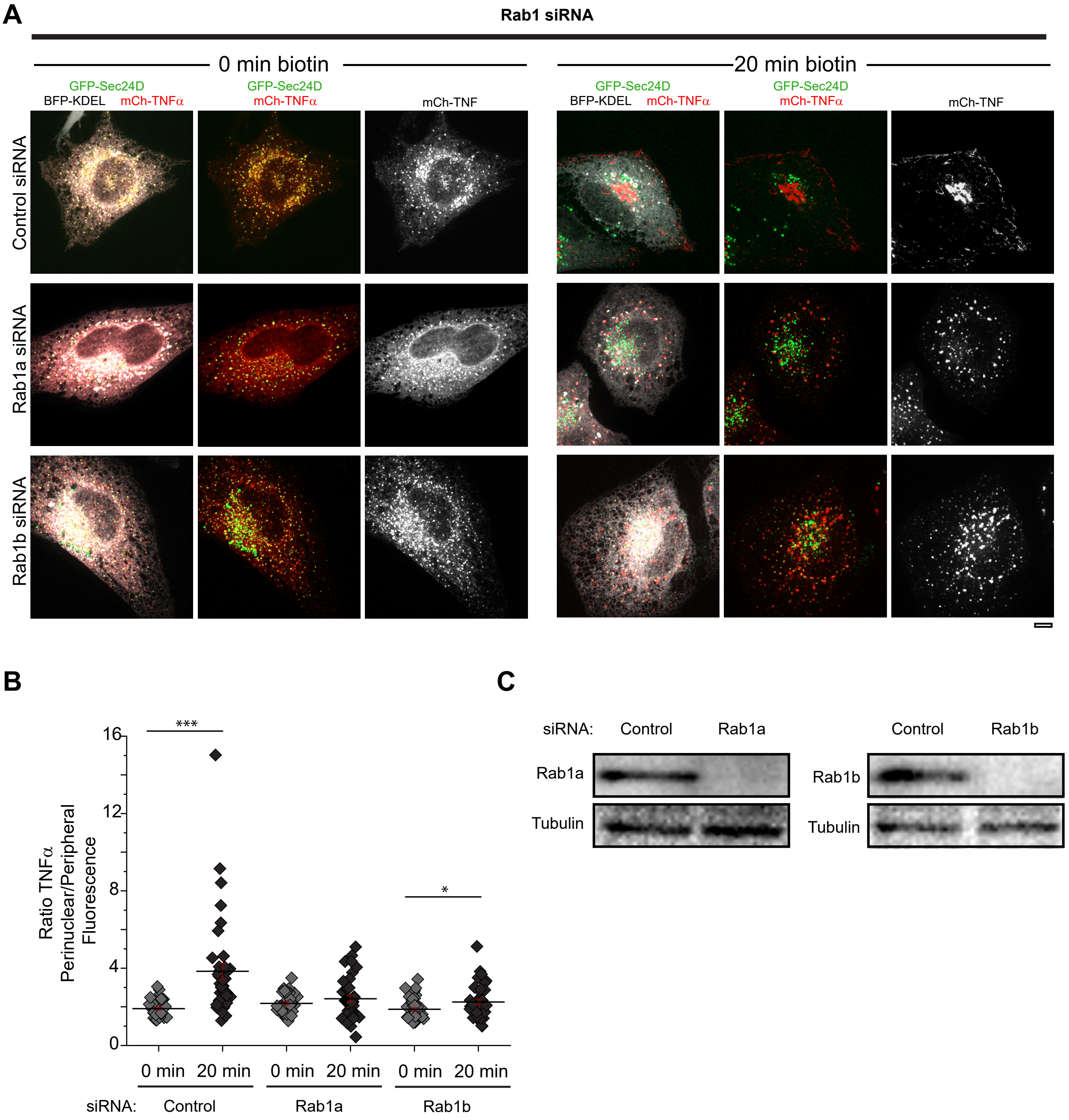

Figure S6: Loss of Rab1 impairs cargo export from the ER.
A. Representative examples of cargo localization before or 20min after biotin treatment in HeLa cells expressing mCh-TNF RUSH (red), GFP-Sec24D (green) and BFP-KDEL (ER, white). Cells belonged in1 of 3 groups, Control, Rab1a or Rab1b siRNA treated cells. B.. Quantification of TNF cargo recruitment to the Golgi was measured by tracking fluorescence intensity measured within a 10 x 10 μm^2 ROI in the perinuclear region versus peripheral ER at 0 min and 20 min following biotin addition in Control, Rab1a or Rab1b siRNA treated cells. Redistribution of cargo to the perinuclear/Golgi region was quantified by plotting the ratio of fluorescence intensity of TNF cargo between the perinuclear and peripheral ROI's. C. Western blot confirmation of protein knockdown for Rab1a and Rab1b. Error bars represent standard error about the mean. Scale bars=5 µm.

Journal of Cell Science • Supplementary information
