# Supplementary material for: Vesicular and uncoated Rab1-dependent cargo carriers facilitate ER to Golgi transport
Source: J Cell Sci. 2020 Jul 24;133(14):jcs239814. doi: 10.1242/jcs.239814 (PMC7390636; doi:10.1242/jcs.239814)
Supplement: Reviewer comments [file joces-133-239814_review_history.pdf]

Vesicular and uncoated Rab1-dependent cargo carriers facilitate ER to Golgi transport

Laura M. Westerate, Melissa J. Hoyer, Michael J. Nash and Gia K. Voeltz
DOI: 10.1242/jcs.239814

Editor: Jennifer Lippincott-Schwartz

Review timeline
Original submission: 29 September 2019
Editorial decision: 16 December 2019
First revision received: 11 March 2020
Editorial decision: 15 June 2020
Second revision received: 17 June 2020
Accepted: 19 June 2020

Original submission

First decision letter

MS ID#: JOCES/2019/239814

MS TITLE: Vesicular and uncoated Rab1-dependent cargo carriers facilitate ER to Golgi transport

AUTHORS: GIA VOELTZ, Laura Westerate, Melissa Hoyer, and Michael Nash
ARTICLE TYPE: Research Article

We have now reached a decision on the above manuscript.

To see the reviewers' reports and a copy of this decision letter, please go to: https://submit-jcs.biologists.org and click on the 'Manuscripts with Decisions' queue in the Author Area. (Corresponding author only has access to reviews.)

As you will see, the reviewers all felt your work was of high quality and significant, but raise a number of criticisms that prevent me from accepting the paper at this stage. They suggest, however, that a revised version should prove acceptable, if you can address their concerns. If you think that you can deal satisfactorily with the criticisms on revision, I would be pleased to see a revised manuscript. We would then return it to the reviewers.

Please ensure that you clearly highlight all changes made in the revised manuscript. Please avoid using 'Tracked changes' in Word files as these are lost in PDF conversion.

I should be grateful if you would also provide a point-by-point response detailing how you have dealt with the points raised by the reviewers in the 'Response to Reviewers' box. Please attend to all of the reviewers' comments. If you do not agree with any of their criticisms or suggestions please explain clearly why this is so.

© 2020. Published by The Company of Biologists under the terms of the Creative Commons Attribution License (https://creativecommons.org/licenses/by/4.0/).

Reviewer 1

Advance summary and potential significance to field

The manuscript “Vesicular and uncoated Rab1-dependent cargo carriers facilitate ER to Golgi 1 transport” by Westrate L. M. et al. report on ER to Golgi transport carriers that bud and translocate to the Golgi in the absence of any of the COPII coat subunits. Also, they report that Rab1 a known molecular switch that regulate ER export does localize in the carriers/vesicles with the cargo. High spatiotemporal fluorescent microscopy is used harnessing the RUSH system. The data is well presented and is of very high quality. The interpretation of the results is also accurate. Although many elements of the presented data were previously demonstrated the authors potentially promote the idea that the COPII heterocomplex is may non act by sorting into vesicles but rather act as a protein extruder selecting and concentrating cargo in a COPII deficient ERES membranes for which budding and translocation occur without the presence of COPII. Yet some issues remain obscure as detailed below especially what is the state of the ERGIC compartment in the theory this manuscript is trying to advance. Also the discussion is very short and does not properly cover the potential significance of the data.

Comments for the author

Primarily they elegantly show that the COPII labeled membrane are continuous with the ER and even in the presence of an ER disrupting agent and their local movement is a result of ER membrane dynamics. The RUSH system is than used to visualize ER export.
Here I have a problem with their arguments on the advantages of the RUSH over the thermoreversible VSVG mutant rendering it “more physiological”: Although it is very popular to criticize the use of the thermoreversible folding mutant VSVG as cargo, the alternative option of the RUSH is not necessarily better.
One should consider that the VSVG temperature block and “utilization” of the secretory pathway is using natural cellular mechanisms such as retention of misfolded protein and trafficking based on a PM targeting signal. The RUSH however is essentially a chimera overexpressed with another (overexpressed) protein (hook) that is held in the ER artificially and so its release mechanism. An indication for the problematic use of the RUSH is that the authors observe the cargo stuck in ERESs. To this end, I would ask them to repeat the experiments with VSVG.
Is there a reference to an effect of temperature on MT dynamics that significantly affects vesicle/carrier trafficking?

Also although they thoroughly reference previous work they should carefully look at (Mezzacasa and Helenius, 2002) and (Dukhovny et al., 2008) and answer how the RUSH is concentrated in ERESs and is still retained in ER without biotin?
The authors further demonstrate that the COPII does not move with the cargo and that the cargo is “extruded” as a term that contradicts uncoating. Perhaps I did not understand the rational but to demonstrate extrusion over coat uncoating the simple data to be provided is to show a time dependent cargo accumulation prior to the budding.
The major weakness of this part is that a lot of the data is a confirmation of many previous observations. Also, the readers are exposed only to the vicinity of ERESs. It would significantly strengthen the manuscript if the structures seen arrive and fuse with the Golgi. Interestingly the literature is full of description of the ERGIC compartment between the Golgi and the ER. Is ERGIC the next target of the vesicles? There are also claims that the ERGIC is very close to the ERESs and that the COPII vesicles travel a short distance and to form it. These theories should be addressed at least in the discussion that is very short and somewhat disappointing. Similar remarks apply to the data concerning Rab1: not much of it is novel but rather more on the confirmatory side.

Minor comment:
1. Is the cytosolic fraction of fluorescently tagged COPII proteins are not visible?
Refs:
Dukhovny, A., Papadopulos, A. and Hirschberg, K. (2008). Quantitative live-cell analysis of microtubule-uncoupled cargo-protein sorting in the ER. J Cell Sci 121, 865-76.
Mezzacasa, A. and Helenius, A. (2002). The transitional ER defines a boundary for quality control in the secretion of tsO45 VSV glycoprotein. Traffic 3, 833-49.

© 2020. Published by The Company of Biologists under the terms of the Creative Commons Attribution License (https://creativecommons.org/licenses/by/4.0/).

Reviewer 2

Advance summary and potential significance to field

This paper by Westrate et al describes an analysis of the trafficking of two fluorescently tagged proteins from the endoplasmic reticulum synchronized using the RUSH system. A major limitation of earlier work was that synchronization of ER exit of cargo was limited to a small number of highly-specialized systems including the ts045 mutant of VSVG and procollagen. This work provides an important validation that RUSH will expand the range of cargoes that can be synchronized to facilitate analysis of ER exit and ER to Golgi trafficking. The studies make use of two tagged cargo proteins: TNF-alpha and Mannosidase II. A detailed analysis is made of their temporal relationship with ERES markers including COPII components, and with Rab1. Overall, the work is well-executed. It is not particularly surprising that Rab1b is associated with ERES continuously during its transit to the Golgi apparatus. However, I am not aware that this has been directly shown. Monetta et al Mol. Biol. Cell 18:2400 (2007)
reported that there were motile Rab1b-tagged structures with no preferred direction of movement. However, their study could not distinguish between structures containing secretory cargo and irrelevent Rab1b-tagged structures, and used a previous generation of microscopes with poorer light sensitivity and time resolution. The sliding of ERES over ER and MTs was also interesting and seems to be both novel and have potential implications for the possible structure of ERES.

Comments for the author

The data in the paper look to be of potentially wide interest. However, as written, the paper is highly descriptive. The standard model that most cell biologists have for ER to Golgi transport to mammalian cells is of COPII vesicles budding from relatively static ERES to merge into VTCs which then move along microtubules to the Golgi apparatus. Some people have argued for alternatives including one in which COPII-coated regions of ER detach to form VTCs more directly. Whether the VTCs are formed entirely de-novo from COPII vesicles, or whether the COPII vesicles fuse with elements of a pre-existing intermediate compartment (e.g., as proposed by Saraste) is not entirely resolved. Some of this is covered in the introduction, but the authors' hypothesis at the beginning of the study, and what they are specifically testing in this work is unclear. What model the authors would favor for ER to Golgi trafficking after completing this study is also unclear? What new has been learned here? How does that favor or modify the authors' hypothesis or model?
What questions were not resolved and will require further study? The brief 15-line discussion is very unhelpful on these points. I believe that with some additional work on the introduction, and especially on the discussion, the overall interest level of the paper could be greatly increased.

Does the fact that ERES can move along ER at these rates have implications for how transport structures can form? Has this been shown for mammalian cells before for COPII-or Sec16 labled structures? The authors should make a statement reflecting the novelty of these observations in the light of the existing literature.

Other points

1. In the Introduction, the authors correctly identify the wide range of cargoes that can be analyzed as a major advantage of the RUSH system. More care is required discussing potential disadvantages of the older VSVG system. Two distinct temperature blocks were used, a 40 C block to hold VSVG in ER and a non-physiological 15 C block to favor accumulation of cargo near ERES. The 15 C block is not used in every study, while the 40 C block, which generally falls within the physiological range of the cells studied is the essential block.

2. As mentioned above, the sliding of COPII punctae along ER should be discussed. Do the authors favor that this is due to sliding of the underlying ER, or that the COPII puntae are moving along the surface of the ER? Even though this represents only a small fraction of COPII structures, this finding appears novel in mammalian cells, and depending on the nature of the sliding might constrain the possible structure of ERES.

© 2020. Published by The Company of Biologists under the terms of the Creative Commons Attribution License (https://creativecommons.org/licenses/by/4.0/).

3. Regarding the sliding and some other statements elsewhere in the paper, I think the term "vesicle" should be used with extreme caution when referring to structures visualized at normal light microscopy resolution. Small clusters of COPII vesicles, COPII buds still attached to ER or patches of COPII under ¼ microns in size would all appear as single punctae under the imaging conditions used. A stronger statement might be possible using additional techniques (correlative light/EM or PALM), but I think would fall outside the major scope of the paper.

4. In the validation of the RUSH system (e.g, Figure 3b,c), the accumulation of TNFa in Golgi looks dramatic. However, the images shown are quite high contrast. There is some apparent TNFa in the Golgi at the zero point, and the quantitication indicates only a 2-fold increase with more at the beginning than for the ER marker (which controls for the concentration of ER in the cell center). Same story for ManII. This could indicate some leakiness. Adding VSVG as an additional control using the standard 40 C block and release would be a good idea since the relative merits of the two systems were extensively discussed.

5. In lines 278-284 there is some commentary relating the experimental data to "Current literature has suggested that COPII vesicles may rapidly shed their coat proteins upon fusion into vesicular tubular complexes (VTCs)." The authors suggest their data falsifies this as they see cargo leaving COPII punctae which lose little COPII fluorescence in the process. However, they propose no alternative explanation, even speculative. I believe this paragraph belongs in the Discussion rather than Results section. Given that Balch and others have claimed to visualize multiple COPII buds in a small area near nascent VTCs using electron microscopy, and that the VTCs may not form instantly, it's unclear to me if the results are really contradictory (although Balch did use temperature blocks and semi-permeabilized cells).

6. In Figure 4a (bottom left) there are some very large tubules containing mCh-TNFalpha. These look like the post-Golgi transport intermediates that have been visualized with VSVG. Can the authors confirm that this is or isn't the case?

7. (minor). Nakano, A. and Muramatsu (line 830 and in the text) is cited with an accent on the second n in Nakano.

Reviewer 3

Advance summary and potential significance to field

In this interesting study Westrate and coworkers employ live-cell imaging to examine the dynamics of cargo and trafficking proteins at ER exit sites (ERES) in three different cell lines. The study provides careful descriptions of these dynamics and suggest an instructive role for Rab1 in COPII-dependent anterograde carrier formation. The RUSH system is employed to partially synchronize cargo exit. There are two key observations. First, the authors see COPII puncta almost exclusively at ERES but not on non-ERES-associated carriers. Second, they report that a dominant-negative Rab1 mutant impairs cargo release at the ERES. These results will add to a longstanding (and recently re-emerging) discussion of what the actual role of COPII is in anterograde carrier formation, nicely build on existing work, and will be of interest to a broad cross section of people working on membrane traffic. The experiments for the most part are descriptive (I do not see that as a shortcoming per se) and done to a high standard, and will contribute to the ongoing discussion of the mechanisms of COPII carrier formation and consumption. However, there are several minor deficiencies in the report, and three significant shortcomings which blunt the impact of the study but which should be readily addressable.

Comments for the author

Major concerns.
1. The authors report that, although post-ERES carriers label with Rab1, ectopic expression of dominant-negative Rab1a N124I or Rab1b N121I variants prevents cargo exit but not concentration at ERES sites (Fig. 6). From these results they infer that carrier egress from the ERES is Rab1-dependent. This interpretation is, in my view, uncertain. The two Rab1 mutants used operate by eliminating a hydrogen bond between Rab1 and the bound guanosine base. This is dominant-

© 2020. Published by The Company of Biologists under the terms of the Creative Commons Attribution License (https://creativecommons.org/licenses/by/4.0/).

negative because the nucleotide-free Rab1 has increased affinity for its nucleotide exchange factor, the TRAPP complex, which poisons the TRAPP GEF activity (e.g., Jones… Segev 08). However, there are multiple forms of TRAPP which take as clients not only Rab1 but Rab 11, as shown by e.g., the Segev and Fromme laboratories. All TRAPP complexes use a single catalytic site, raising the possibility that dominant-negative Rab1 isoforms also deplete Rab11. For these reasons it's important that more selective impairment of Rab1a/b function, e.g., by knockdown CRISPR-mediated deletion, or a combination thereof, be tested. As a less-preferred alternative, the phenotypes arising from dominant-negative Rab11 might be compared to those resulting from dominant-negative Rab1 expression.

2. Key papers are not cited or discussed. These include:
• Moresomme and Riezman 02, who show that yeast Rab1 is needed for protein sorting at ER exit;
• Wagner… Galwitz 87 and Jones… Segev 08, who describe the mechanisms by which N12xI mutations impair function in yeast Rab1;
• Bacon… Ferro-Novick, and Cao…Barlowe 00, which report that defects in yeast Rab1 function impair function mainly at Golgi acceptor compartments, not ER exit sites;
• Shindiapina & Barlowe 10, who show that COPII and ERES are relatively static structures in budding yeast, and Kirk and Ward 07, who review earlier studies showing the same in Pichia pastoris and other eukaryotes.
• Cai… Ferro-Novick 07, who show that in COPII-dependent cell-free budding reactions, the diffusible carrier vesicles retain substantial amounts of COPII subunits (a result also reported in the original COPII paper by Barlowe… Schekman).
• Hanton…Brandizzi 04 and 09, which show that in plants COPII is rather static at ERES, but that these sites spatially coincide with cis-Golgi compartments.

3. The latter observations, in particular, raise the question of the spatial relationship between the ERES as followed in this report, and ERGIC and cis-Golgi markers. At the very least it would be informative to see if impairment of Rab1 function alters the spatial relationship between these compartments and the ERES.

Minor concerns.
4. Line 69: the sentence refers to mammalian cells but the Antonny paper is on yeast COPII.
5. Why were the three cell lines used here chosen?
6. To image for extended periods the authors image at rather low temporal frequencies (typically 0.2 Hz).
This raises the concern that more rapid dynamics might have been missed. Were any pilot studies done at higher frame rates to reduce this concern?
7. The SDCM methods section should include information on tube lens magnification (if non-unity) and (if possible) laser power at the sample plane.
8. Statistical tests appear to be used appropriately but it's not always clear what the error bars indicate.

-Alexey Merz

First revision

<u>Author response to reviewers' comments</u>

Reviewer Response
Dear Dr. Lippincott-Schwartz,
We appreciate the time and effort that you and the reviewers have dedicated to provide valuable feedback on our manuscript “Vesicular and uncoated Rab1-dependent cargo carriers facilitate ER to Golgi transport” for Journal of Cell Science. We have incorporated several changes to reflect most of the suggestions provided by the reviewers which have improved the manuscript. Below is a point-by-point response to the reviewers’ comments and concerns.

© 2020. Published by The Company of Biologists under the terms of the Creative Commons Attribution License (https://creativecommons.org/licenses/by/4.0/).

Comments from Reviewer 1
Comment 1: Here I have a problem with their arguments on the advantages of the RUSH over the thermoreversible VSVG mutant rendering it “more physiological.” To this end, I would ask them to repeat the experiments with VSVG. Is there a reference to an effect of temperature on MT dynamics that significantly affects vesicle/carrier trafficking?
Response: We have accordingly performed experiments with VSVG and have found that similar to the RUSH cargo, VSVG accumulates in COPII enriched foci and following temperature reduction, traffics towards the Golgi unmarked by COPII markers. The data from our VSVG experiments have been incorporated into Figure 4F and supplemental Figure 4C. The discussion of the results can be found on line # 275-289. In addition, we have removed the language in the introduction arguing that RUSH is more physiological and have instead highlighted our use of different methods here to visualize/characterize the dynamics of protein export from the ER.

Comment 2: Also although they thoroughly reference previous work they should carefully look at (Mezzacasa and Helenius, 2002) and (Dukhovny et al., 2008) and answer how the RUSH is concentrated in ERESs and is still retained in ER without biotin?
Response: Thank you for highlighting these manuscripts; we have included a discussion of both of these manuscripts in our introduction/results (line # 222-226 and line # 443-449).

Comment 3: The authors further demonstrate that the COPII does not move with the cargo and that the cargo is “extruded” as a term that contradicts uncoating. Perhaps I did not understand the rational but to demonstrate extrusion over coat uncoating the simple data to be provided is to show a time dependent cargo accumulation prior to the budding.
Response: We originally used “extruded” as description for how cargo appears to exit without COPII fluorescent signal. We appreciate the reviewer’s comment and identification of a potential source of confusion. We have altered the wording and instead highlighted the export and movement of cargo from the ER in an uncoated manner.

Comment 4: The readers are exposed only to the vicinity of ERESs. It would significantly strengthen the manuscript if the structures seen arrive and fuse with the Golgi. Interestingly the literature is full of description of the ERGIC compartment between the Golgi and the ER. Is ERGIC the next target of the vesicles? There are also claims that the ERGIC is very close to the ERESs and that the COPII vesicles travel a short distance and to form it. These theories should be addressed at least in the discussion that is very short and somewhat disappointing. Similar remarks apply to the data concerning Rab1: not much of it is novel but rather more on the confirmatory side.
Response: We have attempted to visualize this process and have tracked cargo events that seem to end in the Golgi region (as in supplemental Figure 5, example 1). We are limited by the fact that the focal plane for monitoring cargo export in the peripheral ER is not the same as that of the cargo once it traffics to the perinuclear region. As such, we are often unable to track cargo all the way to the Golgi. However, throughout the manuscript, we are only monitoring events that are moving towards the Golgi region/ perinuclear region.

Minor Comment 1: Is the cytosolic fraction of fluorescently tagged COPII proteins not visible?
Response: The cytosolic fraction of fluorescently tagged Sec23, 24 and 31 is visible as a general cytosolic stain in the cell however the intensity of signal is far less than that of accumulated fluorescent signal on the peripheral ER. In general, to avoid potential off targets we selected cells with very low expression levels of fluorescently tagged COPII markers which is why the cytosolic fraction is difficult to see.

Comments from Reviewer 2
Comment 1: The standard model that most cell biologists have for ER to Golgi transport to mammalian cells is of COPII vesicles budding from relatively static ERES to merge into VTCs which then move along microtubules to the Golgi apparatus. Some people have argued for alternatives including one in which COPII-coated regions of ER detach to form VTCs more directly. Whether the VTCs are formed entirely de-novo from COPII vesicles, or whether the COPII vesicles fuse with elements of a pre-existing intermediate compartment (e.g., as proposed by Saraste) is not entirely resolved. Some of this is covered in the introduction, but the authors’ hypothesis at the beginning of the study, and what they are specifically testing in this work is unclear. What model the authors would favor for ER to Golgi trafficking after completing this study is also unclear? What new has been learned here? How does that favor or modify the authors’ hypothesis or model? What

© 2020. Published by The Company of Biologists under the terms of the Creative Commons Attribution License (https://creativecommons.org/licenses/by/4.0/).

questions were not resolved and will require further study? The brief 15-line discussion is very unhelpful on these points. I believe that with some additional work on the introduction, and especially on the discussion, the overall interest level of the paper could be greatly increased.
Response: We have added sources to the introduction and have expanded the discussion to address the questions and ideas brought up by reviewer 2. (See line #49-114, 443-489)

Comment 2: In the Introduction, the authors correctly identify the wide range of cargoes that can be analyzed as a major advantage of the RUSH system. More care is required discussing potential disadvantages of the older VSVG system. Two distinct temperature blocks were used, a 40 C block to hold VSVG in ER and a non-physiological 15 C block to favor accumulation of cargo near ERES. The 15 C block is not used in every study, while the 40 C block, which generally falls within the physiological range of the cells studied is the essential block.
Response: We appreciate the reviewer's comment and have adjusted the introduction accordingly. We have also performed experiments related to reviewer 1's feedback that have provided a more complete characterization of available cargo export technologies.

Comment 3: As mentioned above, the sliding of COPII punctae along ER should be discussed. Do the authors favor that this is due to sliding of the underlying ER, or that the COPII puntae are moving along the surface of the ER? Even though this represents only a small fraction of COPII structures, this finding seems novel in mammalian cells, and depending on the nature of the sliding might constrain the possible structure of ERES.
Response: Thank you for this suggestion, we have clarified our interpretation of this data in the discussion (line# 438-443) to highlight our view that COPII co-traffics with the ER as the ER rearranges along microtubules.

Comments 4: Regarding the sliding and some other statements elsewhere in the paper, I think the term "vesicle" should be used with extreme caution when referring to structures visualized at normal light microscopy resolution. Small clusters of COPII vesicles, COPII buds still attached to ER or patches of COPII under ¼ microns in size would all appear as single punctae under the imaging conditions used. A stronger statement might be possible using additional techniques (correlative light/EM or PALM), but I think would fall outside the major scope of the paper.
Response: We agree and have changed "vesicle" to "COPII fluorescent punctum/punctae as we are not able to differentiate between an individual vesicle or cluster of vesicles.

Comments 5: In the validation of the RUSH system (e.g, Figure 3b,c), the accumulation of TNFa in Golgi looks dramatic. However, the images shown are quite high contrast. There is some apparent TNFa in the Golgi at the zero point, and the quantification indicates only a 2-fold increase with more at the beginning than for the ER marker (which controls for the concentration of ER in the cell center). Same story for ManII. This could indicate some leakiness. Adding VSVG as an additional control using the standard 40 C block and release would be a good idea since the relative merits of the two systems were extensively discussed.
Response: We have added VSVG as a control using the standard 40C block and release to provide a more comprehensive look at the protein export from the ER with current available systems. VSVG data has been incorporated into Figures 4 and S4 and discussed in results (line # 266-299).

Comment 6: In lines 278-284 there is some commentary relating the experimental data to "Current literature has suggested that COPII vesicles may rapidly shed their coat proteins upon fusion into vesicular tubular complexes (VTCs)." The authors suggest their data falsifies this as they see cargo leaving COPII punctae which lose little COPII fluorescence in the process. However, they propose no alternative explanation, even speculative. I believe this paragraph belongs in the Discussion rather than Results section. Given that Balch and others have claimed to visualize multiple COPII buds in a small area near nascent VTCs using electron microscopy, and that the VTCs may not form instantly, it's unclear to me if the results are really contradictory (although Balch did use temperature blocks and semi-permeabilized cells).
Response: We have modified that paragraph (line #291-299) to only describe our results and not to discuss the presence or absence of VTCs in cells. In that results paragraph, we have emphasized that we cannot tell if a puncta is one vesicle or a cluster of vesicles. However, it is a clear result that in the events we capture that the cargo signal moves away but the COPII fluorescence signal remains stable at the ER. We have expanded our results section to discuss and emphasize the

© 2020. Published by The Company of Biologists under the terms of the Creative Commons Attribution License (https://creativecommons.org/licenses/by/4.0/).

importance of a stable COPII pool linked to the ER and that COPII does not label the trafficking cargo that moves way from the ER.

Comment 7: In Figure 4a (bottom left) there are some very large tubules containing mCh-TNFalpha. These look like the post-Golgi transport intermediates that have been visualized with VSVG. Can the authors confirm that this is or isn't the case?
Response: The large tubules are very reminiscent of the post-Golgi transport intermediates that have been visualized with VSVG. We did not analyze these larger tubular structures. We focused our analysis with strict criteria, only on cargo that left the ERES in a resolvable puncta.

Comment 8: (minor). Nakano, A. and Muramatsu (line 830 and in the text) is cited with an accent on the second n in Nakano.
Response: Thank you for this correction, we have fixed the citation.

Comments from Reviewer 3
Comment 1: The authors report that, although post-ERES carriers label with Rab1, ectopic expression of dominant-negative Rab1a N124I or Rab1b N121I variants prevents cargo exit but not concentration at ERES sites (Fig. 6). From these results they infer that carrier egress from the ERES is Rab1-dependent. This interpretation is, in my view, uncertain. The two Rab1 mutants used operate by eliminating a hydrogen bond between Rab1 and the bound guanosine base. This is dominant-negative because the nucleotide-free Rab1 has increased affinity for its nucleotide exchange factor, the TRAPP complex, which poisons the TRAPP GEF activity (e.g., Jones… Segev 08). However, there are multiple forms of TRAPP which take as clients not only Rab1 but Rab 11, as shown by e.g., the Segev and Fromme laboratories. All TRAPP complexes use a single catalytic site, raising the possibility that dominant-negative Rab1 isoforms also deplete Rab11. For these reasons it's important that more selective impairment of Rab1a/b function, e.g., by CRISPR medicated knockdown, or a combination thereof, be tested. As a less-preferred alternative, the phenotypes arising from dominant-negative Rab11 might be compared to those resulting from dominant negative Rab1 expression.
Response: We have addressed this concern by coupling the data performed with dominant negative Rab1a/b with data obtained following siRNA knockdown. The new data has been added to the results section (Figure S6, discussed line # 395-399). Briefly, what we found was that cargo recruitment to the Golgi was impaired upon siRNA knockdown of Rab1a or Rab1b. This result is similar to the cargo trafficking impairment we see when using the dominant negative Rab1a(N124I) or Rab1b(N121I).

Comment 2: Key papers are not cited or discussed. These include:
Moresomme and Riezman 02, who show that yeast Rab1 is needed for protein sorting at ER exit; Wagner… Galwitz 87 and Jones… Segev 08, who describe the mechanisms by which N12xI mutations impair function in yeast Rab1; Bacon… Ferro-Novick, and Cao…Barlowe 00, which report that defects in yeast Rab1 impair function mainly at Golgi acceptor compartments, not ER exit sites; Shindiapina & Barlowe 10, who show that COPII and ERES are relatively static structures in budding yeast, and Kirk and Ward 07, who review earlier studies showing the same in Pichiapastoris and other eukaryotes; Cai..Ferro-Novick 07, who show that in COPII-dependent cell-free budding reactions, the diffusible carrier vesicles retain substantial amounts of COPII subunits (a result also reported in the original COPII paper by Barlowe… Schekman); Hanton…Brandizzi 04 and 09, which show that in plants COPII is rather static at ERES.
Response: Thank you for highlighting these references; we have included these papers throughout the manuscript (line #59-63, 179-180, 363-383).

Comment 3: The latter observations, in particular, raise the question of the spatial relationship between the ERES as followed in this report, and ERGIC and cis-Golgi markers. At the very least it would be informative to see if impairment of Rab1 function alters the spatial relationship between these compartments and the ERES.
Response: We agree that this is a very interesting question. By confocal imaging we did not see any significant redistribution or relocalization of COPII exit site markers following Rab1 inhibition. We have added this observation to the manuscript, line 385-387.

Minor Comments from Reviewer 3:
Line 69: the sentence refers to mammalian cells but the Antonny paper is on yeast COPII.

© 2020. Published by The Company of Biologists under the terms of the Creative Commons Attribution License (https://creativecommons.org/licenses/by/4.0/).

Response: This error has been corrected.
Why were the three cell lines used here chosen?
Response: The three mammalian cell lines were chosen for their relative flatness and therefore ease of imaging with a SDCM as well as their transfection efficiency.
To image for extended periods the authors image at rather low temporal frequencies (typically 0.2 Hz). This raises the concern that more rapid dynamics might have been missed. Were any pilot studies done at higher frame rates to reduce this concern?
Response: Pilot studies were performed at lower temporal resolution but we became limited by the sensitivity of detection as exposure levels were lowered to accommodate low temporal frequencies.
The SDCM methods section should include information on tube lens magnification (if non-unity) and (if possible) laser power at the sample plane.
Response: all images were taken using a 100x objective. The method section has been updated to clarify. Lasers were aligned to release at least 12 mW out of the optic cable before entering the confocal head.
Statistical tests appear to be used appropriately but it's not always clear what the error bars indicate.
Response: Figure legends were clarified to indicate whether the error bars represented standard error or standard deviation.

Second decision letter

MS ID#: JOCES/2019/239814

MS TITLE: Vesicular and uncoated Rab1-dependent cargo carriers facilitate ER to Golgi transport

AUTHORS: GIA VOELTZ, Laura Westerate, Melissa Hoyer, and Michael Nash
ARTICLE TYPE: Research Article

I am delighted to say that the reviewers, as well as I, feel your paper is now very much worth publishing in JCS. It is excellent work. The reviewers have suggested that you add three key references related to prior imaging work of secretory cargos. This includes the papers of Luini (collagen), Kreitzer et al., and the BioRxiv paper of Shomron et al.,. Once you have done this, please send the paper back to the JCS office and I will accept it immediately.

Reviewer 1

Advance summary and potential significance to field

This manuscript provides data that may initiate or rather continue a shift of the current dogma that COPII cargo sorting is coupled to vesicle formation. Here it is shown that COPII resides at ERESs and does not participate in the translocation process as does Rab1. Scattered parts of the data have been previously published however here the authors are pushing towards the dogma shift while avoiding some hard questions such as the ERGIC issue as well as what is the significance/meaning of all the published (EM, crystallography permeabilised cells etc.) data on the COPII vesicles.

Comments for the author

Most of my comments were adequately addressed except the issue of what is the status of ERGIC after considering this new data? According to the data inn this manuscript ERGIC is non-existent: COPII accumulates cargo in ERESs followed the budding of membrane vesicles/carriers that translocate on MTs to fuse with the Golgi apparatus. This reviewer agrees that this may actually be the case. Thus, what is the meaning of all the piles of manuscripts relating to the ERGIC. Is ERGIC essentially a large transport carrier? this should be addressed in the discussion as requested previously.

© 2020. Published by The Company of Biologists under the terms of the Creative Commons Attribution License (https://creativecommons.org/licenses/by/4.0/).

I do not know what is the policy of JCS regarding citing manuscripts in BioArchives but a manuscripts that largely overlaps with the data here has been posted about a year ago: Shomron et al. 2019. Uncoating of COPII from ER exit site membranes precedes cargo accumulation and membrane fission. bioRxiv:727107.

Minnor comments: Figure 4F is not labelled with VSVG

Reviewer 2

Advance summary and potential significance to field

The paper is greatly improved and I feel my comments have been adequately addressed. In particular, the context and significance of the findings are well described, and the discussion in particular has been greatly improved. Regarding the mechanisms by which proteins leave ER and the role of Rab1a/b, this paper raises important issues which I think will require future studies to address.

Comments for the author

Minor:

In lines 75-77 the authors state that ts-VSVG is the single cargo that has previously been synchronized to study the secretory pathway. This is true of the overwhelming majority of studies, but there are examples of other strategies. E.g., Luini and coworkers synchronized procollagen release (as mentioned only slightly later in the paper), and Kreitzer and Rodriguez-Boulan used a strategy of microinjection of massive amounts of plasmid DNA to produce rapid synchronized expression of proteins to study sorting to domains of MDCK cells. So, the sentence should be modified slightly.

Second revision

Author response to reviewers' comments

-We have revised our manuscript to include the 3 additional suggested references.
-We have also corrected the labelling on Figure 4F.
-We have modified the sentence about VSVG as suggested by reviewer 2.

Third decision letter

MS ID#: JOCES/2019/239814

MS TITLE: Vesicular and uncoated Rab1-dependent cargo carriers facilitate ER to Golgi transport

AUTHORS: GIA VOELTZ, Laura Westerate, Melissa Hoyer, and Michael Nash
ARTICLE TYPE: Research Article

I am happy to tell you that your manuscript has been accepted for publication in Journal of Cell Science, pending standard ethics checks.

© 2020. Published by The Company of Biologists under the terms of the Creative Commons Attribution License (https://creativecommons.org/licenses/by/4.0/).
